# RNA-seq transcriptome profiling of pigs’ liver in response to diet with different sources of fatty acids

**DOI:** 10.3389/fgene.2023.1053021

**Published:** 2023-01-25

**Authors:** Simara Larissa Fanalli, Bruna Pereira Martins da Silva, Julia Dezen Gomes, Mariah Castro Durval, Vivian Vezzoni de Almeida, Gabriel Costa Monteiro Moreira, Bárbara Silva-Vignato, Juliana Afonso, Felipe André Oliveira Freitas, James Mark Reecy, James Eugene Koltes, Dawn Koltes, Dorian Garrick, Luciana Correia de Almeida Regitano, Júlio Cesar de Carvalho Balieiro, Gerson Barreto Mourão, Luiz Lehmann Coutinho, Heidge Fukumasu, Severino Matias de Alencar, Albino Luchiari Filho, Aline Silva Mello Cesar

**Affiliations:** ^1^ Faculty of Animal Science and Food Engineering, (FZEA), University of São Paulo, São Paulo, Brazil; ^2^ Animal Science Department, Luiz de Queiroz College of Agriculture (ESALQ), University of São Paulo, Piracicaba, Brazil; ^3^ Department of Animal Science, College of Veterinary Medicine and Animal Science, Goiânia, Brazil; ^4^ Unit of Animal Genomics, GIGA Medical Genomics, University of Liège, Liège, Brazil; ^5^ Embrapa Pecuária Sudeste, São Carlos, Brazil; ^6^ Animal Science Department, Iowa State University, Ames, IA, United States; ^7^ AL Rae Centre for Genetics and Breeding, Massey University, Hamilton, New Zealand; ^8^ College of Veterinary Medicine and Animal Science, University of São Paulo, Pirassununga, Brazil

**Keywords:** RNA-Seq, metabolic diseases, transcriptome, metabolic disease, neurodegenerative diseases, liver, pig

## Abstract

Pigs (*Sus scrofa*) are an animal model for metabolic diseases in humans. Pork is an important source of fatty acids (FAs) in the human diet, as it is one of the most consumed meats worldwide. The effects of dietary inclusion of oils such as canola, fish, and soybean oils on pig gene expression are mostly unknown. Our objective was to evaluate FA composition, identify changes in gene expression in the liver of male pigs fed diets enriched with different FA profiles, and identify impacted metabolic pathways and gene networks to enlighten the biological mechanisms’ variation. Large White male pigs were randomly allocated to one of three diets with 18 pigs in each; all diets comprised a base of corn and soybean meal to which either 3% of soybean oil (SOY), 3% canola oil (CO), or 3% fish oil (FO) was added for a 98-day trial during the growing and finishing phases. RNA sequencing was performed on the liver samples of each animal by Illumina technology for differential gene expression analyses, using the R package DESeq2. The diets modified the FA profile, mainly in relation to polyunsaturated and saturated FAs. Comparing SOY vs*.* FO, 143 differentially expressed genes (DEGs) were identified as being associated with metabolism, metabolic and neurodegenerative disease pathways, inflammatory processes, and immune response networks. Comparing CO vs*.* SOY, 148 DEGs were identified, with pathways related to FA oxidation, regulation of lipid metabolism, and metabolic and neurodegenerative diseases. Our results help explain the behavior of genes with differential expression in metabolic pathways resulting from feeding different types of oils in pig diets.

## 1 Introduction

Lipids have a variety of different functions within the body and are a class of molecules that are present in all types of cells (Nakamura and Nara, 2003; Cesar et al., 2016). Different types of vegetable oils affect the FA profile and differ in their cholesterol-lowering ability. For example, vegetable oils can exhibit cholesterol-lowering effects (Saedi et al., 2017). Fatty acids (FAs) are derived from both animal fats, such as tallow and poultry fat, and plant oils, such as canola, flaxseed, sunflower, and corn (Moghadasian and Shahidi, 2017; Gomes et al., 2022).

Pigs (*Sus scrofa*) produce pork which is one of the most consumed meats worldwide, making them an important livestock species and an important source of protein and FA in the human diet (USDA, 2022). Pork is rich in saturated fatty acids (SFAs), monounsaturated fatty acids (MUFAs), and polyunsaturated fatty acids (PUFAs). Oleic acid, omega-9 (OA, C18:1 cis 9; n-9); linoleic acid, omega-6 (LA, C18:2 cis 9, 12; n-6); and α-linolenic acid, omega-3 (ALA, C18:3 cis-9, 12, 15; n-3) are some of the unsaturated fatty acids that bring benefits to human health (Zhang et al., 2016).

The liver is a central metabolic organ and plays a key role in gene expression regulated by FAs, with PUFAs being the main FA regulators of hepatic lipogenic gene expression (Jump et al., 1993; Mater et al., 1998). In pigs, PUFA synthesis, *de novo* lipid and cholesterol synthesis, and FA oxidation mainly occur in the liver (Xing et al., 2019). Alterations in the metabolic balance of lipid pathways in the liver and dysregulation of energy control lead to pathological conditions such as diabetes, obesity, and metabolic syndrome (Shimizu et al., 2015; Park and Han, 2018). In addition, some nutrients and hormonal and neuronal signals are responsible for regulating the metabolism of lipids, glucose, and amino acids in the liver (Rui, 2014). However, the effects on pig gene expression of dietary inclusion of different oils such as those present in canola, fish, or soybean oils are mostly unknown. Thus, the findings of this study could help to better understand the impact of the different dietary FAs on the liver transcriptome of pigs as an animal model and their impact on biological processes such as metabolic pathways related to lipid metabolism.

PUFA, ALA, docosahexaenoic acid (DHA), and eicosapentaenoic acid (EPA) are essential FAs for human physiology, as the intake of these FAs is related to a lower risk of stroke and coronary heart disease (Jakobsen et al., 2009; Baker et al., 2016; Moghadasian and Shahidi, 2017). PUFAs are responsible for the activity of the immune system and are related to the regulation of eicosanoid synthesis (Szostak et al., 2016). MUFAs, which are present in olive and canola oils, are synthesized in the human body and help in the prevention of cardiovascular diseases (Guasch-Ferré et al., 2014; Moghadasian and Shahidi, 2017).

Understanding the transcriptome of organisms and their tissues is revolutionized by the RNA-sequencing technique (RNA-Seq), which provides levels of transcripts and their isoforms more precisely than other methods (Wang et al., 2009). Pigs are considered model animals for scientific studies on humans because some aspects of their physiology are very similar to those of humans (Groenen et al., 2012). The present study evaluated the differential expression in the liver of pigs fed diets containing different FA profiles and identified the main metabolic pathways impacted.

## 2 Materials and methods

### 2.1 Ethics approval

All experimental procedures involving animals are in accordance with the requirements of the Animal Care and Use Committee of Luiz de Queiroz College of Agriculture (University of São Paulo, Piracicaba, Brazil; protocol: 2018.5.1787.11.6 and number CEUA 2018-28) and follow ethical principles in animal research, according to the Guide for the Care and Use of Agricultural Animals in Agricultural Research and Teaching (Fass, 2010).

### 2.2 Animals and sample collection

In this study, we selected 54 purebred Large White male pigs. The sires and dams that originated the population were of the Large White breed. We used three sires and 32 dams to keep inbreeding below 14%. The animals were genotyped for the halothane mutation (*RYR1* gene) according to Fujii et al. (1991), and all halothane homozygous negative (NN) were selected for this. The animals at 71 ± 1.8 days of age were allocated to one of three dietary treatments in a design with six replicate pens per treatment and three pigs per pen. Each pen was equipped with a three-hole dry self-feeder and a nipple drinker, which allowed *ad libitum* access to food and water throughout the experimental period. All the pigs were immunocastrated through the administration of two 2-mL doses of Vivax^®^ (Pfizer Animal Health, Parkville, Australia) on days 56 and 70 of the experiment (Almeida et al., 2021), in accordance with the manufacturer’s recommendations.

The experimental diets consisted of corn–soybean meal growing–finishing diets, supplemented with either 3% soybean oil (SOY), 3% canola oil (CO), or 3% fish oil (FO) and were modified according to the growth and finishing phases. Canola oil is an important source of OA, and SO is rich in LA, while FO is an important source of DHA (C22:6; n-3) and EPA (C20:5; n-3) (Almeida et al., 2021). The diets were formulated to have similar metabolizable energy content (3.36 Mcal/kg) and were formulated to meet or exceed requirements (Rostagno, 2011). The diets were formulated to have a similar level of digestible energy. No antibiotic growth promoters were used, and all diets were provided in a mash meal form; details of the animals and diets of this study are described in Supplementary Tables S1–S4, which were adapted from our previous study (Almeida et al., 2021). The average values for the initial body weights of the animals were 28.78 kg for the SOY group, 29.56 kg for the CO group, and 28.10 kg for the FO group (*p*-value of 0.86). After 98 days of trial, all pigs were slaughtered, with an average final body weight of 132.13 kg (CO group), 136.63 kg (SOY group), and 133 kg (FO group) (*p*-value of 0.09). The right lobe of the liver samples were collected and quickly excised, snap-frozen in liquid nitrogen, and then stored at −80°C until further analyses (Almeida et al., 2021).

### 2.3 Fatty acid profile

The FA profile determination was performed from the total lipid isolated from 100 g of the liver samples, and a more complete description of the analyses can be found in Almeida et al. (2021). Briefly, FA were determined using the cold extraction method (Bligh and Dyer, 1959) in accordance with the procedure outlined by AOCS (2004; Method AM 5-04).

### 2.4 Statistical analyses

Statistical analyses to detect differences in the FA profile of the liver between the diets were performed using the MIXED procedure of the SAS statistical software (SAS Institute Inc., Cary, NC, v. 9.4), where a mixed model was fit using restricted maximum likelihood (REML) methodology. In the model, the block effects were fit as random effects and the dietary treatments as fixed effects. The UNIVARIATE procedure (v. 9.4) was used to test for departure from a normal distribution with homogeneity of residuals for each of the variables. Diagnostics of the density distribution of the studentized residual of the model were made using the Shapiro–Wilk test (SAS v. 9.4). Differences were declared significant based on the Tukey test when *p*-values  ≤0.05.

### 2.5 RNA extraction, library preparation, and sequencing

Total RNA was extracted from tissue samples using the RNeasy^®^ Mini Kit (QIAGEN, Hilden, Germany) according to the manufacturer’s instructions. Total RNA quantification, purity, and integrity were evaluated by the NanoDrop 1000 and Bioanalyzer. RNA integrity numbers (RINs) of the samples were between 7.8 and 10.0 (Supplementary Table S5). All samples presented an RIN greater than or equal to 7.8. From the total RNA of each sample, 2 µg was used for library preparation according to the protocol described in the TruSeq RNA Sample Preparation Kit v2 Guide (Illumina, San Diego, CA). The Agilent Bioanalyzer 2100 (Agilent, Santa Clara, CA, United States) was used to calculate average library size, and the libraries were quantified using quantitative PCR with the KAPA Library Quantification Kit (KAPA Biosystems, Foster City, CA, United States). Quantified samples were diluted, labeled by barcoding, and pooled to be run in different lanes (five pools of all 36 samples each) using the TruSeq DNA CD Index Plate (96 indexes, 96 samples, Illumina, San Diego, CA, United States). All samples were sequenced across five lanes of a sequencing flow cell using the TruSeq PE Cluster Kit v4-cBot-HS kit (Illumina, San Diego, CA, United States) and were clustered and sequenced using HiSeq 2500 equipment (Illumina, San Diego, CA, United States) with a TruSeq SBS Kit v4-HS (200 cycles) according to the manufacturer’s instructions. All the sequencing analyses were performed at the Genomics Center in the Animal Biotechnology Laboratory at ESALQ of the USP, Piracicaba, São Paulo, Brazil.

### 2.6 Quality control and alignment

The quality of the raw RNA-Seq reads after trimming was checked with FastQC software version 0.11.8 (http://www.bioinformatics.bbsrc.ac.uk/projects/fastqc/). Subsequently, the sequencing adaptors and low-complexity reads were removed in the initial data-filtering step by Trim Galore 0.6.5 software (https://www.bioinformatics.babraham.ac.uk/projects/trim_galore). Reads with a Phred score higher than 33 and a length higher than 70 nucleotides were kept after trimming. Reads were then aligned with the pig reference genome *Sus Scrofa* 11.1, which is available at Ensembl [http://www.ensembl.org/Sus_scrofa/Info/Index]. The abundance (*read counts*) of mRNAs for all annotated genes was calculated using STAR-2.7.6a (Dobin and Gingeras, 2015; Fanalli et al., 2022a; 2022b). Gene expression levels were normalized using counts scaled by the total number of reads (CPM—counts per million) using the R package DESeq2 (Love et al., 2014).

### 2.7 Differentially expressed genes and functional enrichment analysis

Differentially expressed genes (DEGs) between the pairwise comparisons of different diets (CO vs*.* SOY, SO vs*.* FO, and CO vs*.* FO) were identified using the R package DESeq2, available at Bioconductor open-source software for bioinformatics, using a multi-factor design (Love et al., 2014). Prior to statistical analysis, the read count data were filtered as follows: 1) unexpressed genes were genes with zero counts for all samples; 2) very lowly expressed genes were genes with less than one read per sample on average; and 3) rarely expressed genes were genes that were not present in at least 50% of the samples. Unexpressed, very lowly expressed, and rarely expressed genes were all removed from the analysis. The three sires were used as a fixed factor in the multi-factor model. The cut-off approach performed to identify the DEGs was the methodology of Benjamini and Hochberg (1995), which was used to maintain the false discovery rate (FDR) at 10% (Cesar et al., 2016; Fanalli et al., 2022b). The normalized read counts for the DEG were correlated to the fatty acid content of the liver samples by calculating the Pearson correlation coefficient (Pearson’s r) by using the PROC CORR procedure in SAS.

The functional enrichment analysis was performed using MetaCore software (Clarivate Analytics, 2022) to identify the pathway maps from the list of DEGs. The functional enrichment analysis of DEGs (FDR <0.10) was performed to obtain comparative networks by “analysis of a single experiment” using *Homo sapiens* genome annotation as the background reference and standard parameters of MetaCore software v. 21.4 build 70700. The filters used were metabolic maps: energy metabolism, lipid metabolism, and steroid metabolism; cardiovascular diseases: atherosclerosis; regulation of metabolism; nutritional and metabolic diseases; and nervous system diseases. For more detailed definitions of pathway maps, see https://portal.genego.com/legends/MetaCoreQuickReferenceGuide.pdf.

## 3 Results

### 3.1 Fatty acid profile

The composition of the FA deposited was different between treatments. EPA, DHA, and consequently total n-3 PUFA were higher in the FO group (*p*-value <0.01) than in the CO and SOY groups with no difference observed between the CO and SOY groups. On the other hand, a higher amount of total MUFA and myristic acid (*p*-value <0.05) was observed in the CO and SOY groups compared to the FO group. The n-6:n-3 ratio was lower in the FO group than in the CO and SOY groups, with no difference observed between the CO and SOY groups (*p*-value <0.01). The lowest atherogenic index was observed in the CO group than in the FO and SOY groups, with no difference between the FO and SOY groups, but in reference to the PUFA:SFA ratio (*p*-value <0.05), CO and FO showed higher percentages of tissue deposition than the SOY group. The lowest concentration of total PUFA was identified in the SOY group than in the FO and CO groups with no difference between the FO and CO groups (*p*-value <0.01). Concentrations of palmitic acid, stearic acid, palmitoleic acid, LA, ALA, SFA, and total n-6 PUFA were similar among all diets (see Table 1).

**TABLE 1:** Effects of dietary treatments on the fatty acid profile deposited in the liver of male pigs.

Fatty acid (%)	Dietary treatment			Pooled SEM^2^	*p-*value
	CO	FO	SOY		
Saturated fatty acid (SFA)					
Myristic acid (C14:0)	0.91^12^	0.78^1^	0.98^2^	0.06	0.02
Palmitic acid (C16:0)	21.96	21.61	22.98	0.43	0.06
Stearic acid (C18:0)	23.52	23.92	21.28	1.30	0.28
Monounsaturated fatty acid (MUFA)					
Palmitoleic acid (C16:1)	0.78	0.85	0.93	0.07	0.30
Oleic acid (C18:1 n-9)	24.40^12^	23.08^1^	27.78^2^	1.27	0.02
Polyunsaturated fatty acid (PUFA)					
Linoleic acid (C18:2 n-6)	24.96	22.76	23.64	0.65	0.06
Alpha-linolenic acid (C18:3 n-3)	1.24	1.05	1.17	0.10	0.36
Eicosapentaenoic acid (C20:5 n-3, EPA)	0.66^1^	1.88^2^	0.27^1^	0.19	<0.01
Docosahexaenoic acid (C22:6 n-3, DHA)	1.69^1^	4.09^2^	0.98^a^	0.39	<0.01
Total SFA	45.14	46.31	45.24	1.14	0.69
Total MUFA	25.17^12^	23.92^1^	28.71^2^	1.30	0.02
Total PUFA	28.55^1^	29.77^1^	26.06^2^	0.62	<0.01
Total n-3 PUFA^3^	3.59^1^	7.01^2^	2.42^1^	0.53	<0.01
Total n-6 PUFA^4^	24.96	22.76	23.64	0.65	0.06
PUFA:SFA ratio^5^	0.64^12^	0.65^1^	0.58^2^	0.02	0.03
n-6:n-3 PUFA ratio^6^	8.79^1^	4.67^2^	9.90^1^	0.70	<0.01
Atherogenic index^7^	0.44^1^	0.50^2^	0.51^2^	0.01	<0.01

^1^ Pigs (*n* = 54) were fed a corn–soybean meal diet enriched with 3% canola oil (CO), 3% fish oil (FO), or 3% soybean oil (SOY). Values represent the least square means of 18 pigs/treatment.

^2^ SEM = standard error of the least square means.

^3^ Total n-3 PUFA = {[C18:3 n-3] + [C20:5 n-3] + [C22:6 n-3]}.

^4^ Total n-6 PUFA = C18:2 n-6.

^5^ PUFA:SFA ratio = total PUFA/total SFA.

^6^ Σ n-6/Σ n-3 PUFA ratio.

^7^ Atherogenic index = (4 × [C14:0]) + (C16:0)/[total MUFA] + [total PUFA]), where brackets indicate concentrations (Ulbricht and Southgate, 1991).

^a-b^Within a row, values without a common superscript differ (*p*-value ≤0.05) using Tukey’s method.

### 3.2 Sequencing data and differential expression analysis

The average numbers of total reads sequenced from the liver samples before and after filtering for the CO group were 35,201,462 and 34,736,732; for the soybean oil (SOY) group, 34,078,903 and 33,610,858; and for the FO group, 34,296,605 and 33,801,914, respectively. General mapping statistics are shown in Supplementary Table S5.

A total of 148 DEGs (FDR <0.10) were identified in the CO vs*.* SOY comparison, where 108 were downregulated (log2-fold change ranging from −4.71 to −0.29) and 40 were upregulated (log2-fold change ranging from 0.24 to 2.36) in the CO group compared to the SOY group (Table 2). For the SOY vs*.* FO comparison, 143 DEGs (FDR <0.10) were identified, where 87 were downregulated (log2-fold change ranging from −3.94 to −0.20) and 56 were upregulated (log2-fold change ranging from 0.28 to 1.94) in the SOY group compared to the FO group (Table 2). The CO vs*.* FO comparison did not show any DEG. Supplementary Table S6 presents genes and the log2-fold change found between the comparisons. Supplementary Figure S1 shows the volcano plot of log2-fold change (*x*-axis) vs*.* −log10FDR-corrected *p*-value (*y*-axis) from the DEG analysis for the liver tissue (A) CO vs*.* SOY and (B) SOY vs*.* FO.

**TABLE 2:** Differentially expressed genes found in the liver tissue of pigs fed with different oils in the diet.

Comparison^a^	DEG^b^	log2FC^c^	DEG^b^	log2FC^c^	Total DEG^b^
	Downregulated		Upregulated		
CO *vs*. SOY	108	−4.71 to −0.29	40	0.24 to 2.36	148
CO *vs*. FO	—	—	—	—	0
SOY *vs*. FO	87	−3.94 to −0.20	56	0.28 to 1.94	143

^a^ Comparison: CO, canola oil; SOY, soybean oil; and FO, fish oil.

^b^ Differentially expressed genes.

^c^ log2-fold change.

**FIGURE 1:**
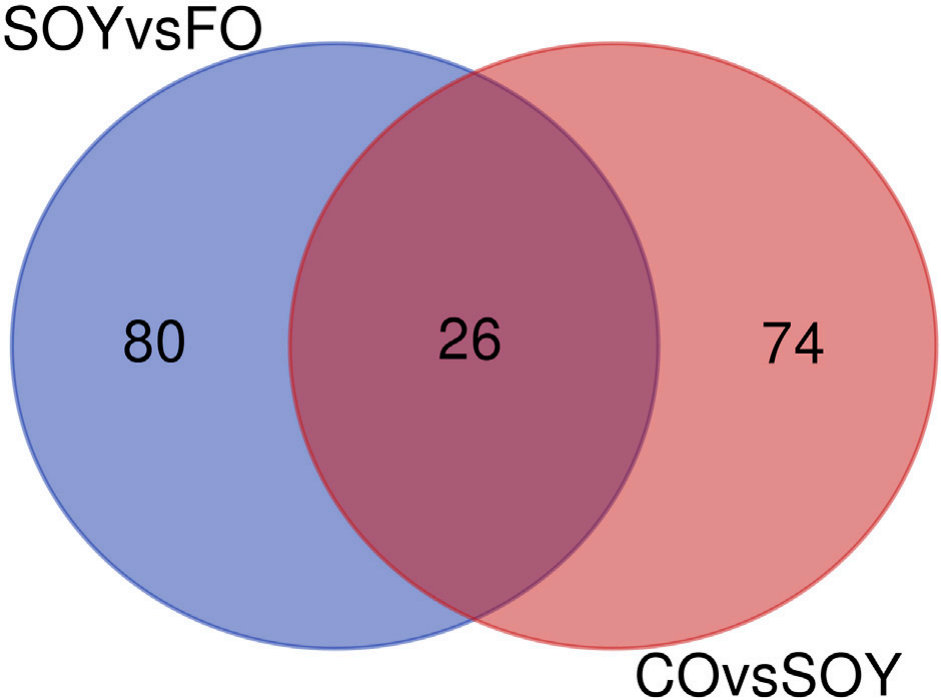
Venn diagram of the comparison of 3.0% soybean oil vs*.* 3.0% fish oil (SOY vs*.* FO) and 3.0% canola oil vs. soybean oil (CO vs. SOY).

We identified 26 common genes between CO vs*.* SOY and SOY vs*.* FO (Figure 1; Table 3). Among them, c*ytochrome P450 family 7 subfamily A member 1* (*CYP7A1;* log2-fold change −2.77) was downregulated in the CO and SOY groups. On the other hand, *phospholipase A2 group IID* (*PLA2G2D;* log2-fold change +1.65) was upregulated in the CO and SOY groups.

**TABLE 3:** Common differentially expressed genes between the CO vs*.* SOY and SOY vs*.* FO comparisons.

DEG^b^			DEG^b^		
Downregulated			Upregulated		
*Gene*	*CO* vs*. SOY* ^a^	*SOY* vs*. FO* ^a^	*Gene*	*CO* vs*. SOY* ^a^	*SOY* vs*. FO* ^a^
*CYP2*B*22*	*−2.45*	*−2.18*	*CD180*	*+0.74*	*+0.89*
*NGEF*	*−0.60*	*−0.65*	*GAS2L1*	*+0.46*	*+0.48*
*TECTB*	*−2.22*	*−1.78*	*CRELD2*	*+0.52*	*+0.85*
*CRY2*	*−0.86*	*−0.67*	*GALK1*	*+0.69*	*+0.65*
*DZANK1*	*−0.55*	*−0.55*	*PLA2G2D*	*+1.43*	*+1.65*
*ABCA8*	*−0.80*	*−0.79*	*DERL3*	*+0.97*	*+0.84*
*GPR1*	*−1.20*	*−0.95*			
*PCBP4*	*−0.52*	*−0.41*			
*ZNF277*	*−0.43*	*−0.38*			
*B3GNT4*	*−1.91*	*−1.39*			
*DBP*	*−1.57*	*−1.27*			
*PIPOX*	*−1.12*	*−0.98*			
*SMAD3*	*−0.32*	*−0.37*			
*CCDC90B*	*−0.42*	*−0.50*			
*CYP7A1*	*−2.31*	*−2.77*			
*RBBP9*	*−0.49*	*−0.45*			
*PRDM10*	*−0.99*	*−0.86*			
*ND6*	*−0.93*	*−0.92*			
*CAST*	*−0.48*	*−0.52*			
*KDM1B*	*−0.48*	*−0.47*			

^a^ Comparison: CO, canola oil; SOY, soybean oil; and FO, fish oil (log2-fold change).

^b^ Differentially expressed genes.

The correlation analysis among the DEG and the fatty acid content of liver samples among the different diets is shown in Supplementary Tables S7, S8, where, in general, the magnitude of the correlation coefficient among gene expression and the fatty acid profile (phenotypes) was moderate to low.

### 3.3 Functional enrichment analysis for differential expression between CO and SOY

In the enriched signaling pathways (*p*-value <0.10; Table 4), we identified genes such as *mitochondrial-encoded NADH dehydrogenase* 5 (*ND5 MTND5*) and *mitochondrial-encoded NADH dehydrogenase 6* (*ND6 MTND6*) that are present in the “*CREB1*-dependent transcriptional deregulation pathway in Huntington’s disease” (Supplementary Figure S2).

**TABLE 4:** Pathway maps from the list of differentially expressed genes (FDR 10%) in the liver of male pigs fed with different oil sources found using MetaCore software (*p*-value <0.10).

Pathway maps	*p-*value	DEG
CREB1-dependent transcription deregulation in Huntington’s disease	0.01	*MTND6* and *MTND5*
Regulation of lipid metabolism_FXR-dependent negative feedback regulation of bile acid concentration	0.01	*CYP2B6* and *CYP7A1*
Triacylglycerol biosynthesis in obesity and diabetes mellitus type II	0.07	*MOGAT2*
Huntington-dependent transcriptional deregulation in Huntington’s disease	0.08	*Ephexin*
Fatty acid omega oxidation	0.09	*CYP4A11*
Putative pathways of oleic acid sensing in the ventromedial hypothalamus in obesity (rodent model)	0.09	*CD36*
Role of adipose tissue hypoxia in obesity and type 2 diabetes	0.09	*CD36*

**FIGURE 2:**
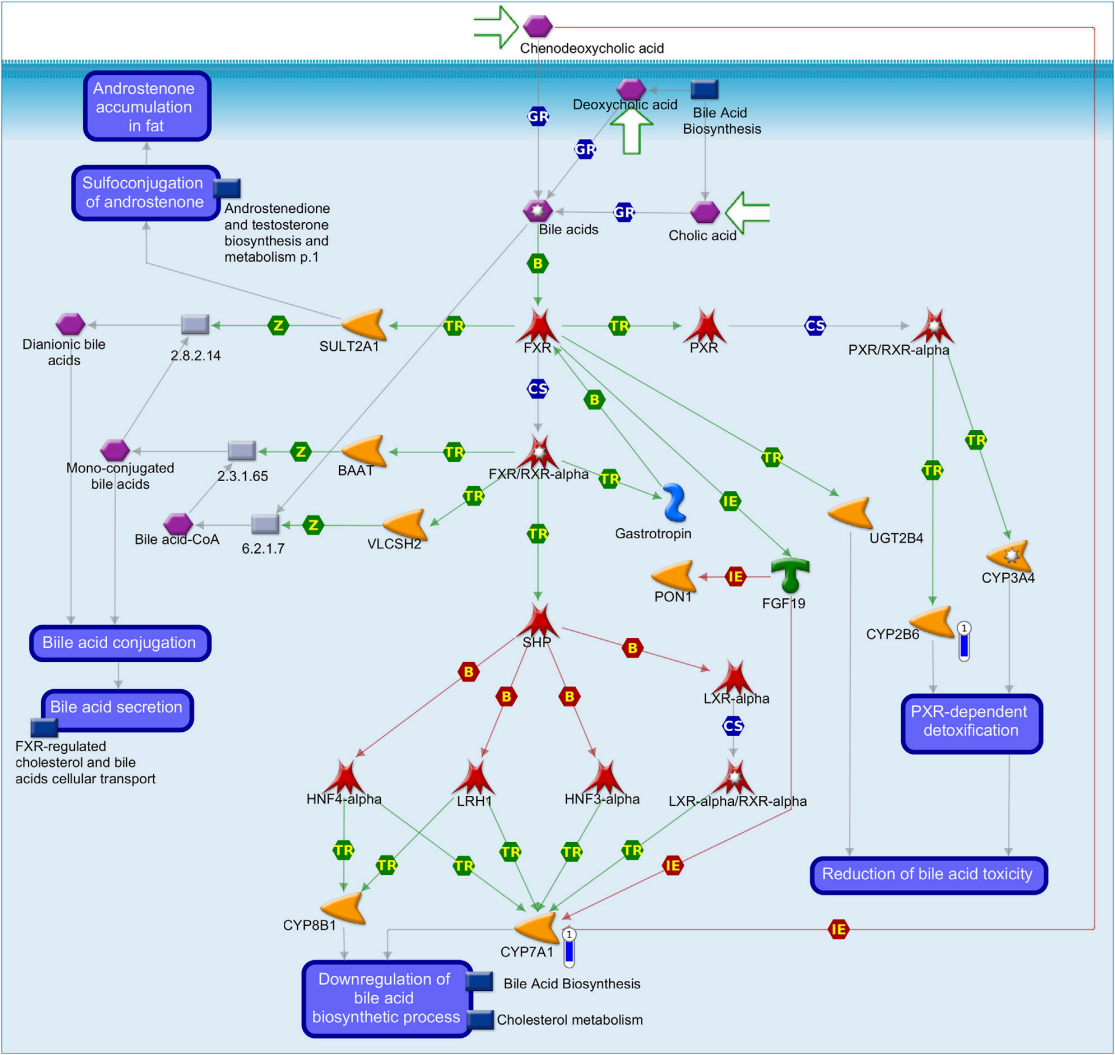
Pathway map representing the regulation of lipid metabolism_FXR-dependent negative feedback regulation of bile acid concentration using MetaCore software (*p*-value <0.10) based on the list of differentially expressed genes (FDR 10%) in the liver tissue of pigs fed with different oils in the diet (3.0% canola oil and 3.0% soybean oil). The blue thermometer indicates that the DEG is downregulated (log2-fold change −2.31 and −2.45) in the diet with 3.0% of canola oil (CO). Green arrows indicate positive interactions, and gray arrows indicate unspecified interactions.

**FIGURE 3:**
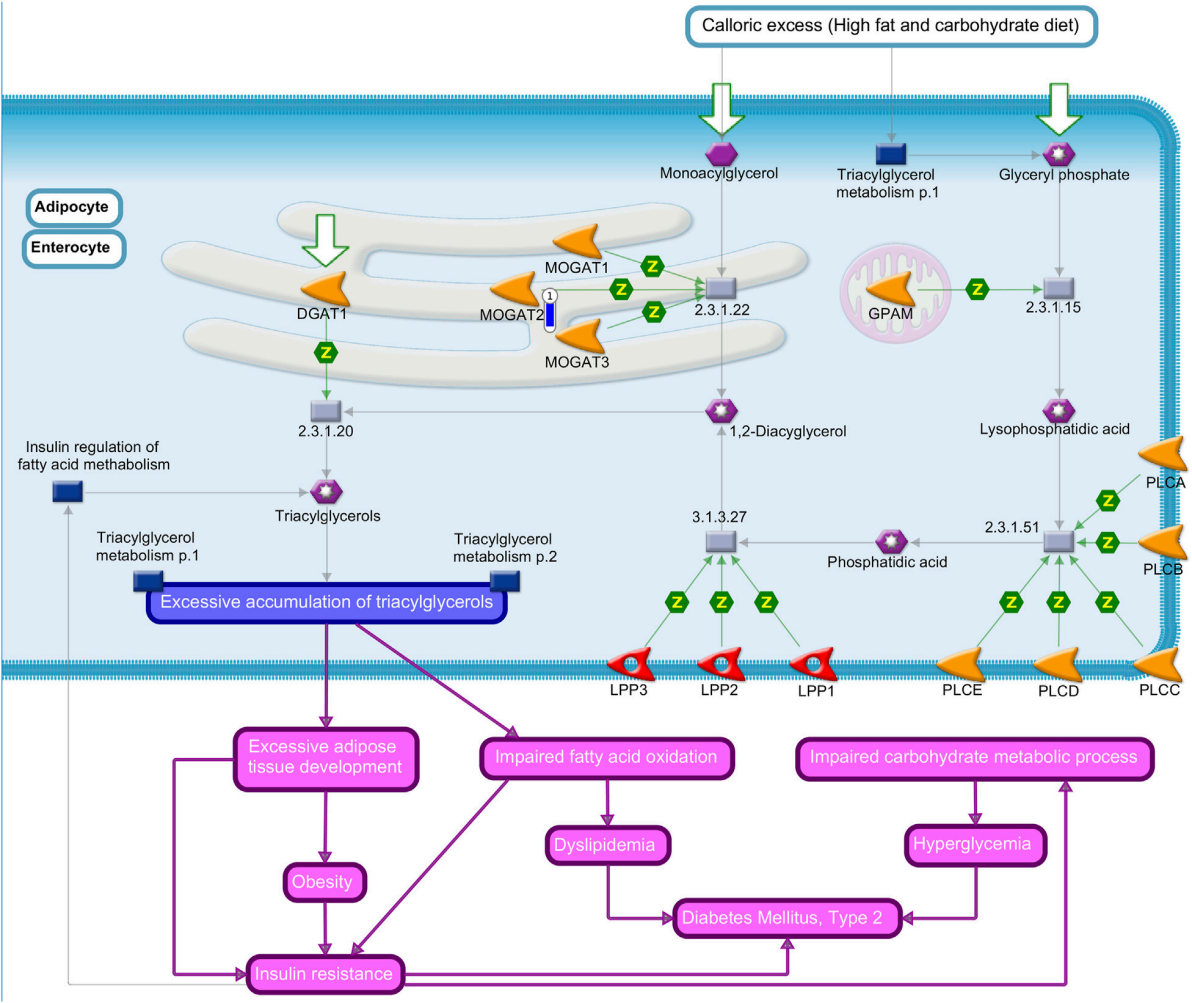
Triacylglycerol biosynthesis in obesity and the diabetes mellitus type II pathway map by MetaCore software (*p*-value <0.10) from the list of differentially expressed genes (FDR 10%) in the liver tissue of pigs fed with different oils in the diet (3.0% canola oil and 3.0% soybean oil). The blue thermometer indicates that the DEG is downregulated (log2-fold change −4.72) in the diet with 3.0% of CO. Purple lines indicate increases in diseases. Green arrows indicate positive interactions, and gray arrows indicate unspecified interactions.

The *cytochrome P450 family 7 subfamily A member 1* (*CYP7A1*) and *cytochrome P450 family 2 subfamily B member* 6 (*CYP2B6*) are related to the “regulation of lipid metabolism *FXR*-dependent negative feedback regulation of bile acid concentration” (Figure 2); *monoacylglycerol O-acyltransferase 2* (*MOGAT2*) was found in the “triacylglycerol biosynthesis in obesity and diabetes mellitus type II” pathway (Figure 3); the neuronal guanine nucleotide exchange factor *NGEF/Ephexin* gene in the “Huntington-dependent transcriptional deregulation pathway in Huntington’s disease” (Supplementary Figure S3); the *cytochrome P450 family 4 subfamily A member* 11 (*CYP4A11*) in “fatty acid omega oxidation” (Supplementary Figure S4); and the *cluster of differentiation 36* (*CD36*) that participates in “putative pathways of oleic acid sensing in the ventromedial hypothalamus in obesity (rodent model)” (Supplementary Figure S5) and the “role of adipose tissue hypoxia in obesity and type 2 diabetes” (Supplementary Figure S6).

Herein, they were identified as DEGs in the liver of pigs fed CO vs*.* SOY with lower expression (log2-fold change −2.31 and −2.45) in pigs of the CO group and enriched in the “lipid metabolism FXR-dependent negative feedback regulation of bile acid concentration” pathway.

*MOGAT2* was identified as the lowest expressed gene (log2-fold change −4.72) in pigs of the CO group present in the “triacylglycerol biosynthesis in obesity and diabetes mellitus type II” (Figure 2).

*CYP4A11* exhibited lower expression in the diet containing CO (log2-fold change −1.25) than in the SOY diet and was enriched in the “fatty acid omega oxidation” pathway.

*NGEF* was identified as a downregulated gene (log2-fold change −0.6) in the CO group. The *NGEF* appears as *Ephexin* in the “Huntington-dependent transcriptional deregulation in Huntington’s disease” pathway. The *CD36* gene was identified as having the lowest expression (log2-fold change −0.48) in the group receiving a diet with CO and being enriched in the “putative pathways of oleic acid sensing in the ventromedial hypothalamus in obesity (rodent model)” and the “role of adipose tissue hypoxia in obesity and type 2 diabetes” pathways.

**FIGURE 4:**
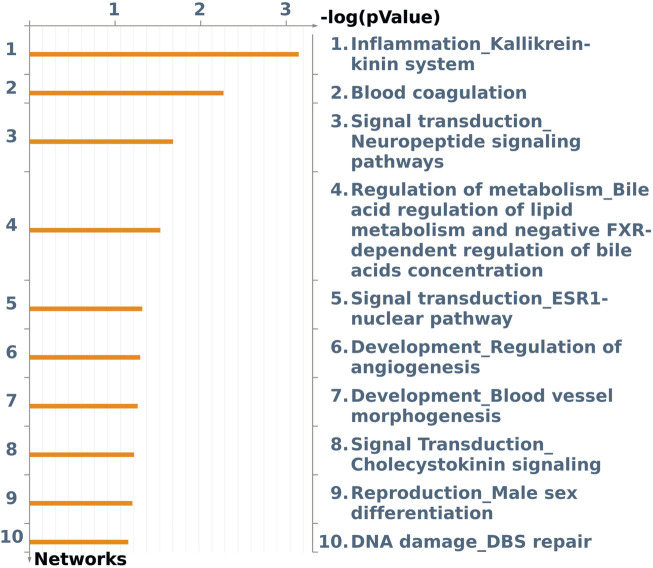
Top 10 enriched networks identified using MetaCore software applied to the list of differentially expressed genes (FDR 10%) in the liver tissue of male pigs fed with different oils (3.0% canola oil and 3.0% soybean oil).

To observe the interactions of DEGs in gene networks, an analysis of process networks was performed using MetaCore software (Figure 4). The identified networks were related to inflammatory processes, metabolism, and neuropeptide signaling. Among the networks obtained, we identified (*p*-value <0.10) the “inflammation_kallikrein–kinin system” (*p*-value 7.143E-04) in which the DEG *kininogen 1* (*KNG1*) (log2-fold change −0.65) is involved; the “signal transduction_neuropeptide signaling” pathway (*p*-value 2.230e-2) presented some DEGs in the Galpha(i)-specific peptide GPCR group, such as the *neuropeptide Y receptor Y1* (*NPY1R*) (log2-fold change −1.09) and the “development/regulation of angiogenesis” pathway (*p*-value 5.475e-2), presenting the DEG *SMAD family member 3* (*SMAD3*) (log2-fold change −0.32) and Galpha(i)-specific peptide GPCR such as *NPY1R* (log2-fold change −1.09).

### 3.4 Functional enrichment analysis for differential expression between SOY and FO

**FIGURE 5:**
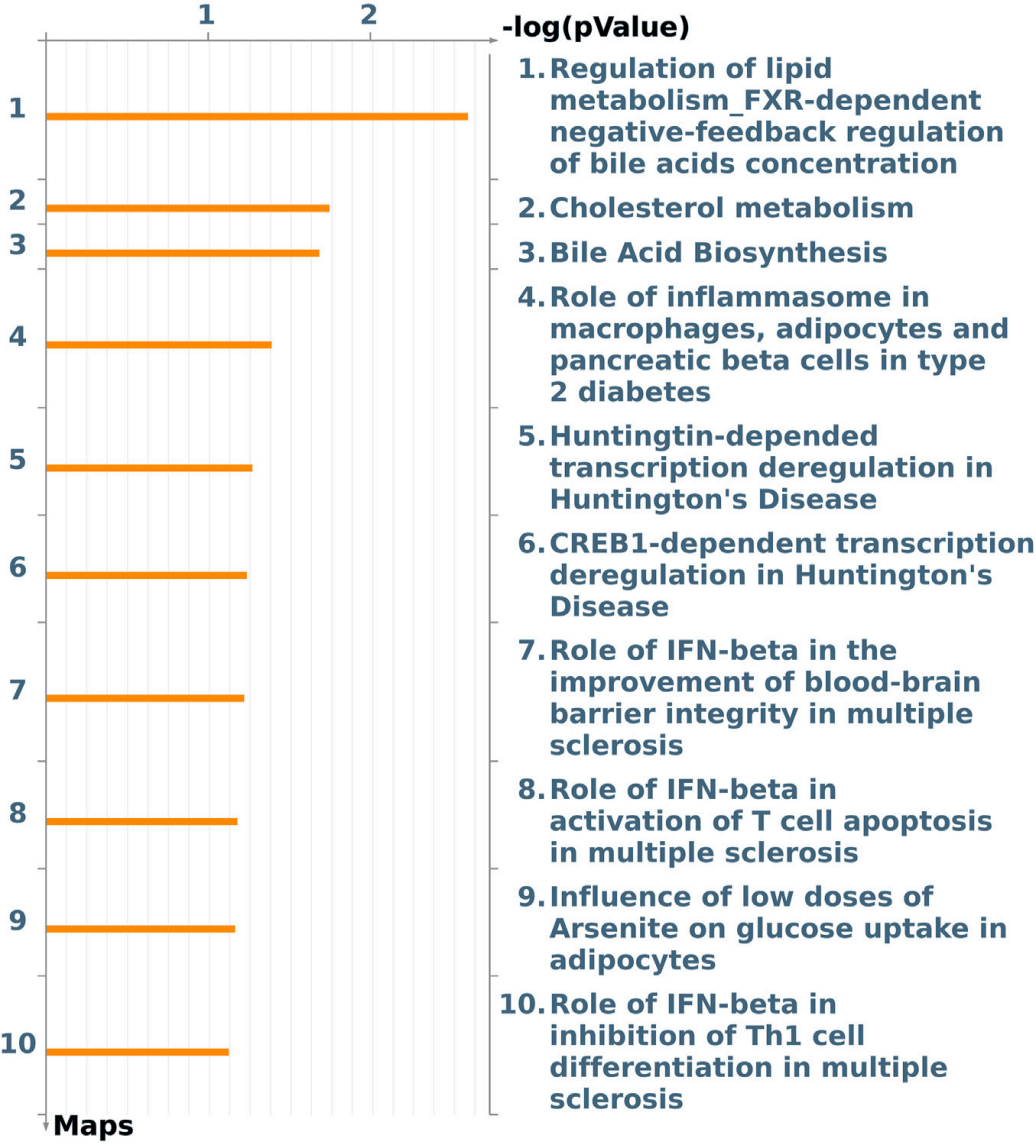
Pathway maps from the list of differentially expressed genes (FDR 10%) in the liver of male pigs fed with different oil sources (3.0% soybean oil vs*.* 3.0% fish oil) detected using MetaCore software (*p*-value <0.10).

The enriched pathway maps (Figure 5) obtained (*p*-value <0.10) were “regulation of lipid metabolism FXR-dependent negative feedback regulation of bile acid concentration” (Supplementary Figure S7) (*p*-value 2.500E-03, log2-fold change −2.18 *CYP2B6* DEG and *CYP7A1* DEG with log2-fold change −2.78); “cholesterol metabolism” (Supplementary Figure S8) (*p*-value 1.851E-02, log2-fold change −0.38 *ACOX2* DEG and *CYP7A1*); “bile acid biosynthesis” (Supplementary Figure S9) (*p*-value 2.140E-02, *ACOX2* and *CYP7A1* DEG); the “ role of inflammasome in macrophages, adipocytes, and pancreatic beta cells in type 2 diabetes” (Supplementary Figure S10) (*p*-value 4.253E-02, log2-fold change +0.49 *IL-18* DEG); and “Huntington-dependent transcriptional deregulation in Huntington’s disease” (Supplementary Figure S11) (*p*-value 5.632E-02, *Ephexin* DEG).

*CYP7A1* is enriched in other pathways with the gene *acyl-CoA oxidase 2 (ACOX2)*, which was identified as a DEG and has lower expression in the group fed a diet enriched with soybean oil. The *CYP7A1* and *ACOX*2 DEGs were enriched in “cholesterol metabolism” and “bile acid biosynthesis” pathways, highlighting the importance of their relationship to bile acids.

**FIGURE 6:**
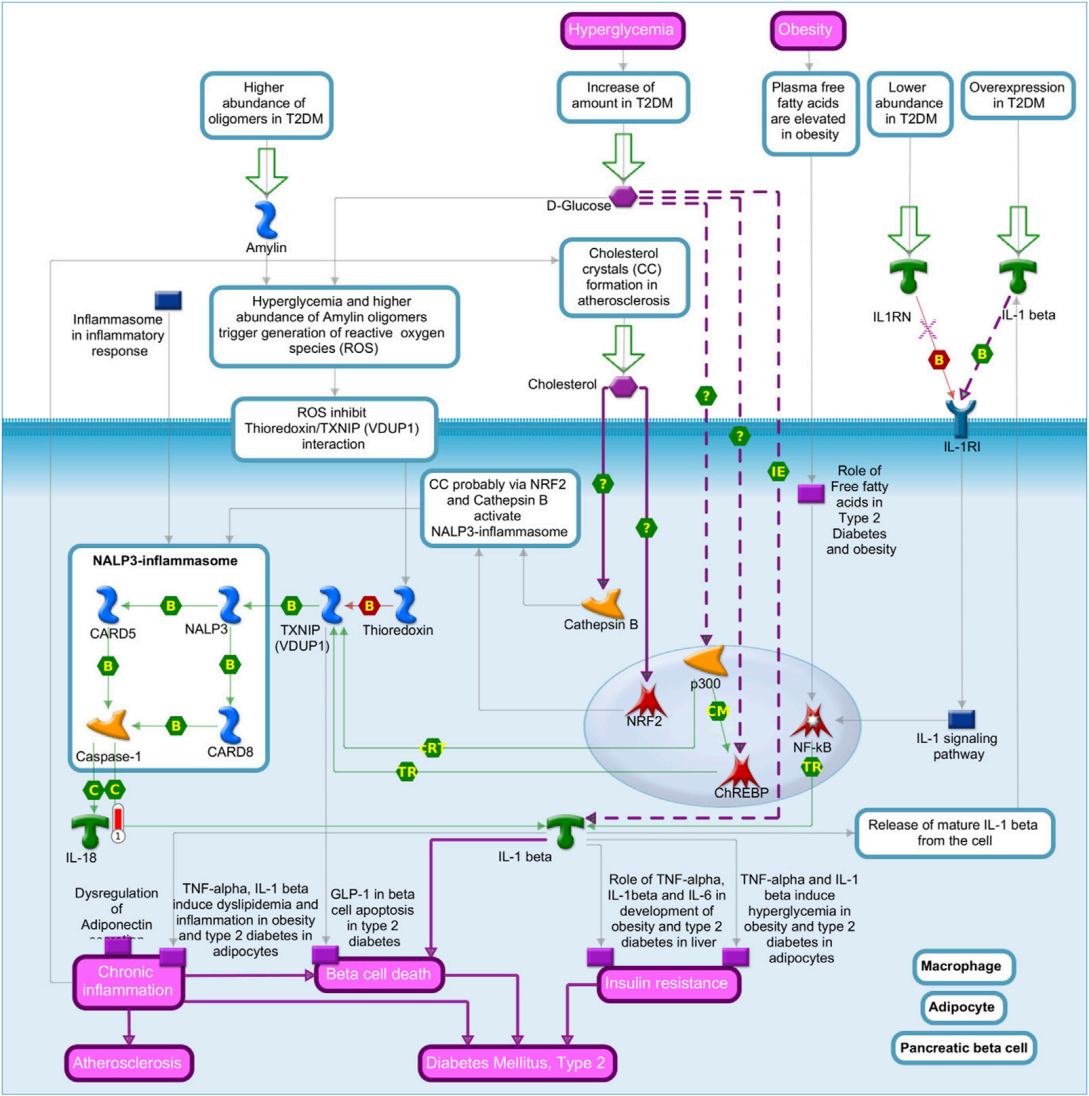
Role of the inflammasome in macrophages, adipocytes, and pancreatic beta cells in the type 2 diabetes pathway map based on MetaCore software (*p*-value <0.10) from the list of differentially expressed genes (FDR 10%) in the liver tissue of pigs fed with different oils in the diet (3.0% soybean oil and 3.0% fish oil). The blue thermometer indicates that the DEG is downregulated in the diet with 3.0% soybean oil (SOY). The large green arrows indicate the path to start. Green arrows indicate positive interactions, and gray arrows indicate unspecified interactions. Links in purple indicate emerging and enhanced (dotted) diseases.

*Interleukin-18* (*IL-18*) showed higher expression in the liver of pigs fed with the SOY diet and was enriched in the “role of the inflammasome pathway in macrophages, adipocytes, and pancreatic beta cells in type 2 diabetes” (Figure 6). Another enriched pathway that *IL-18* is involved in is the “role of *IFN-beta* in inhibition of Th1 cell differentiation in multiple sclerosis” (Supplementary Figure S12).

Another DEG identified was *NGEF* which exhibited lower expression (log2-fold change −0.65) in pigs fed with the SOY diet and was enriched within the “Huntington-dependent transcriptional deregulation pathway in Huntington’s disease” as *Ephexin. MTND6* presented a lower expression (log2-fold change −0.92) in the SOY group, in addition to being enriched in the “*CREB1-*dependent transcriptional deregulation pathway in Huntington’s disease”.

*Kinesin Family Member 3B (KIF3B)* was identified in our study as having a lower expression (log2-fold change −0.53) in the liver of pigs fed with the SOY diet. It participates in the “influence of low doses of arsenite on glucose uptake in adipocytes” pathway.

In addition to these analyses, a process network analysis (Table 5) was performed to assist in the understanding of the enriched pathways through interactions in gene networks that are related to the change of oil in the diet. We highlight here the pathways of “regulation of bile acid metabolism,” “regulation of lipid metabolism,” “negative *FXR*-dependent regulation of bile acid concentration” and pathways related to signal transduction, sodium transport, and neurophysiological processes.

**TABLE 5:** Process networks from the list of differentially expressed genes (FDR 10%) in the liver tissue of male pigs fed with different oil sources found using MetaCore software (*p*-value <0.1).

Process networks	*p*-value	DEG
Signal transduction_ESR1 nuclear pathway	1.081E-02	*BCAS3*, *SMAD3*, *CYP7A1*, and *DLC1 (dynein LC8a)*
Regulation of metabolism_bile acid regulation of lipid metabolism and negative FXR-dependent regulation of bile acid concentration	3.351E-02	*CYP2B6* and *CYP7A1*
Transport_sodium transport	6.506E-02	*SLC5A9*, *SN1*, and *SN2*
Reproduction_male sex differentiation	7.348E-02	Olfactory receptor, *SMAD3*, and calpastatin
Neurophysiological process_olfactory transduction	7.476E-02	Olfactory receptor
Cytoskeleton_cytoplasmic microtubules	7.926E-02	*KIF3B* and *DLC1 (dynein LC8a)*
Cardiac development BMP and TGF-beta signaling	8.165E-02	*MSX1* and *SMAD3*
Signal transduction_androgen receptor nuclear signaling	9.267E-02	*SMAD3* and *NRIP*

## 4 Discussion

In this study, we identified the differential expression of genes in the livers of pigs fed diets containing different FA profiles. Despite the similarities in FA composition of CO and SOY diets, for example, OA, EPA, total SFA, and total MUFA compositions, transcriptome differences were found, revealing genes and pathways relevant to lipid processes and diseases. The composition of FA in tissues is not only affected by the ingestion of FA but also by the genotype of the animal, body mass, and mainly, the deposition of FA in the tissues is related to specific rates of deposition, synthesis, and desaturation (Poklukar et al., 2020; Sarri et al., 2022). More studies are needed to understand the incorporation of dietary FA in pig tissues (Sarri et al., 2022). Although the deposition of FA in the different tissues is modulated by the diet, mainly in monogastric species, this deposition is dependent on the interconversions between them, limiting the impact of dietary additions (Wood et al., 2008). The decrease in MUFA deposition has already been reported in pigs to be little affected by dietary concentrations as reviewed by Wood et al. (2008), and *de novo* fatty acid synthesis may dominate fatty acid profiles in some circumstances.

The correlation analysis showed moderate-to-low Pearson correlation coefficients among normalized read counts and the fatty acid content in liver tissue among the comparisons between the diets. The OA and total MUFA content correlated positively with the *NCEH1* gene, and this gene has already been related to lipid metabolism in a muscle transcriptome study (Puig-Oliveras et al., 2014). Because of the fatty acid deposition complexity as phenotype, it involves several genes and biological processes related with so many interactions among genes, so to determine the linear correlation between gene expression and phenotype is still a challenge. Other alternatives to measure his correlation were proposals such as Spearman or Pearson correlation with maximal information component analysis (MICA) combined with the interaction component model or distance correlation as incorporated in weighted gene co-expression network analysis (WGCNA) (Hou et al., 2022). Our results corroborate with previous studies (Darwiche and Struhl, 2020; Hou et al., 2022), where the authors suggested that a co-expression analysis is a better approach to reveal the correlation among the DEGs and fatty acids studied herein.

When comparing the CO vs*.* SOY diets, we found differentially expressed genes present in a signaling pathway related to *CREB1*. The TF CREB1 is critical for lipid synthesis and triacylglycerol accumulation (Yao et al., 2020). This TF is involved in the regulation of adipocyte differentiation, lipogenesis, and insulin activity in adipose tissues (Klemm et al., 2001). *Heat shock protein 9* of 70 kDa (*GRP75*) is involved in the delivery of *CREB1* to mitochondria. Interruption of *CREB1* activity in mitochondria leads to decreased expression of mitochondrial-encoded *MTND6* and *MTND5*, downregulating complex I-dependent mitochondrial respiration and consequent mitochondrial dysfunction and neuronal cell death in Huntington’s disease (Lee et al., 2005). The *MTND6* gene may be related to reactive oxygen species (ROS) production and transcriptional regulation of *CREB1* directly, in which mitochondrial *CREB1* binds to the promoter of *MTND6* and promotes its expression (Lee et al., 2005). The NADH-ubiquinone oxidoreductase chain such as ND5 and ND6 has been associated with the development of important roles in liver tissue in purebred Landrace female pigs in high-feed and low-feed efficiency groups. These genes were highly expressed in the liver tissues of both groups (Wang et al., 2022). In humans, there are studies related to MT-ND genes associated with mutations in complex I subunits (Danhelovska et al., 2020). In our experimental conditions, we found the differentially expressed genes *ND6* and *ND5* that are downregulated in the CO group.

The *CYP2B6* and *CYP7A1* genes in the CO vs*.* SOY comparison (both downregulated in the CO group) were enriched in the “regulation of metabolism_bile acid regulation of lipid metabolism and negative FXR-dependent regulation of bile acid concentration” process network, with a lower expression in the CO group. Bile acids in high concentrations are toxic (Jung et al., 2006). The CYP2B6 enzyme is involved in the reduction of bile acid toxicity, and the downregulation of the CYP7A1 enzyme is involved in the decrease in bile acid biosynthesis and is found in the endoplasmic reticulum of hepatocytes (Jung et al., 2006; Guevara-Ramírez et al., 2022). The CYP7A1 protein is of great importance in the metabolism of steroids, cholesterol, and other lipids (Guevara-Ramírez et al., 2022). The liver is the organ that expresses all of the necessary enzymes for the bile acid synthesis. The degradation of cholesterol to form bile acids can be through *CYP7A1* by the classical pathway, the main biosynthetic pathway of bile acids in humans or through mitochondrial *CYP7A1* by the alternative (acidic) pathway due to the production of acid intermediates (Norlin et al., 2000; Chiang and Ferrell, 2020). In a study evaluating the liver transcriptome in pigs from extreme groups of the intramuscular composition of fatty acids, *CYP7A1* was identified as a downregulated DEG in the low (L) group in contrast to the high (H) group, being considered as a candidate gene mainly related to its role in the processes of fatty acid metabolism in pigs in the liver (Ramayo-Caldas et al., 2012). The authors emphasize the importance of studies that identify how different types of FA control gene expression through a direct examination of the effect of each FA on fatty acid composition (Ramayo-Caldas et al., 2012). Szostak et al. (2016) suggested that with an increased contribution of omega-3 fatty acids in the liver, there is an association with negative regulation of lipid metabolism and increased β-oxidation. The authors identified *CYP7A1* as a DEG in a study of the effect of an enriched diet based on linseed and rapeseed oil supplementation on the porcine liver transcriptome, corroborating with Ramayo-Caldas et al. (2012) and our study in which a diet with a higher amount of n-3 (CO group) showed downregulated expression of *CYP7A1*. In addition, PPAR-*alpha* showed altered expression in our study, being an important indicator of the oxidation-metabolic state and intracellular transport of FA, similar to the findings of Ramayo-Caldas et al. (2012) and Szostak et al. (2016). Other studies have related *CYP7A1* to lipid metabolism (Wang et al., 2020; Lyu et al., 2022), which highlights the importance of our study when verifying that the diet with oils modified the expression in pigs. In humans, CYP2B6 is the only hepatic isoform of CYP2B that plays a role in the metabolism of numerous xenobiotic and endobiotic compounds (Heintz et al., 2022). Cholesterol and dietary fats modulate *CYP7A1* activity and gene expression in humans, but the exact mechanism by which fatty acids can regulate CYP7A1 expression remains to be elucidated (Fu et al., 2011). Thus, the genes identified as belonging to the SOY dietary group may show a better relationship with detoxification, while genes from the CO dietary group showed a decrease in bile acid synthesis. Again, the importance of enriched DEGs that are involved in regulatory pathways and networks related to bile acids that regulate glucose and lipid metabolism in hepatocytes is noted.

The modification of the diets with the oils resulted in changes in *CD36* gene expression, which may be involved in relevant pathways, related to inflammatory processes and diseases. *CD36* binds to long-chain FAs (LCFAs) and can function in the transport and/or as a regulator of fatty acid transport (Stelzer et al., 2016) and a negative regulator of angiogenesis and inflammation. *CD36* is ubiquitously expressed, being present in cells such as hepatocytes, adipocytes, cardiac and skeletal myocytes, and specialized epithelia of the breast, kidney, and intestine (Silverstein and Febbraio, 2009). According to Silverstein and Febbraio (2009), *CD36* is related to the pathogenesis of diseases such as atherosclerosis and Alzheimer’s disease. The LCFAs circulate in the blood, and small portions of LCFA enter the brain, where FA metabolism within the ventromedial hypothalamic region (VHM) can function as a sensor for nutrient availability (Le Foll et al., 2009). The detection of hypothalamic OA is impaired in animal models for obesity due to an increase in proteins that reverse the esterification of OA and other effects that can decrease acyl-CoA (oleoyl-CoA), thus contributing to an increase in food intake and glucose production (Lane et al., 2008). *CD36* was also enriched in the “role of adipose tissue hypoxia in obesity and type 2 diabetes” pathway, and it is a shared target of *LXR*, *PXR*, and *PPAR-gamma* (Yin et al., 2009). Decreased expression of *FATP1* and *CD36* may result in the inhibition of FA absorption and increased accumulation of free FA. The mechanisms and effects of this pathway result in a decrease in adiponectin, and an underexpression in obesity leads to insulin resistance (Chen et al., 2006; Yin et al., 2009). In addition to hypertrophy and adipocyte death that can be caused by inhibition linked to PPAR-gamma, this increase in apoptosis of adipose tissue leads to macrophage infiltration in adipose tissue and a release of stored FA (Weisberg et al., 2003; Levin et al., 2011). Furthermore, the *CD36* gene in humans is currently being investigated as a potential target in some diseases, such as cardiovascular disease, metabolic syndrome, obesity, and cancer (Niculite et al., 2019). Thus, in this study, we identified *CD36* modified by dietary oils, and in the CO group, a low expression of *CD36* was identified when compared to SOY.

There was no differential gene expression observed between the CO and FO diets, which was expected based on the fatty acid profile of the liver of animals fed with both diets (Table 1). We observed a significant difference in the amounts of EPA and DHA and consequently in the n-3, n-6:n-3 ratio, and atherogenic index. However, the increased EPA and DHA deposition was not sufficient to differentially modify the liver transcriptome as found in other studies (Zhang et al., 2019). Adequate intake of both n-3 and n-6 contributes to the long-term reduction of LDL cholesterol (Lima et al., 2022). The CO and FO oils are rich in PUFA, with a high content of OA, which are used as an alternative to improve the lipid profile of meat products (Lima et al., 2022). Canola and fish oils showed a beneficial immunoregulatory effect and controlled atherosclerosis in a study with cells (macrophages derived from human blood monocytes) treated with fatty oils of different omega-6/omega-3 ratios (Subash-Babu and Alshatwi, 2018).

In the comparison between SOY and FO in the cholesterol metabolism pathway, *ACOX2* participates through the activating effect on the catalysis mechanism of a bile acid intermediate (Van Veldhoven et al., 1994). *ACOX2* encodes an enzyme related to the metabolism of branched chain FA and bile acid intermediates (Zhang et al., 2021). Thus, cholesterol metabolism can be decreased with the expression of *CYP7A1* and *ACOX2* in the SOY group in relation to FO. In a muscle transcriptome study using pigs with extreme values of FA composition performed by Puig-Oliveras et al. (2014), *ACOX2* was enriched related to lipid metabolism with functions related to oxidation, synthesis, and insulin resistance. *ACOX2* deficiency has been reported in human liver-related diseases (Zhang et al., 2021).

Concerning the SOY vs*.* FO comparison, DEGs were enriched in relevant signaling pathways, such as IL-18. The *IL-18* DEG is a classical member of the IL-1 superfamily of cytokines and is a pro-inflammatory, pro-atherogenic cytokine (Aso et al., 2003). IL-18 binds to its specific receptor, triggering intercellular responses that result in the activation of NF-κB and inflammatory processes (Somm and Jornayvaz, 2022). Pro-IL-18 is produced by various cells, but initially, the main source of IL-18 described was the Kupffer cell (liver-resident macrophage), which plays an important pathophysiological role in health and disease (Yasuda et al., 2019; Somm and Jornayvaz, 2022).

In a study with an obese mini-pig model fed a diet with high sugar content (33% sugar) and fat (10% lard), IL-18, together with TNF-α, IL-6, leptin, and IL -1β, increased significantly (Zhu et al., 2019). High levels of this cytokine were identified in a study with humans presenting with type 2 diabetes (Aso et al., 2003), showing possible participation in disease development (Thorand et al., 2005). Also, in humans, elevated circulating levels of *IL-18*, a product of the activation of the nucleotide binding and oligomerization domain-like receptor family pyrin domain-containing 3 (*NLRP3*) inflammasome, have been identified in patients with metabolic syndrome (Yasuda et al., 2019). However, *IL-18* alone does not exhibit a pro-inflammatory effect when other cytokines are low in expression (Yasuda et al., 2019).

*IL-18* was enriched in the “role of the inflammasome in macrophages, adipocytes, and pancreatic beta cells in type 2 diabetes” pathway by MetaCore. *IL-18* may be related to metabolic syndrome and its consequences, that is, it may be involved in the set of risk factors that identify a population at increased risk for developing metabolic syndrome, which encompasses type 2 diabetes and cardiovascular diseases (Trøseid et al., 2010). Furthermore, *IL-18* has atherogenic properties through effects on *IL-6*, *TNF-α*, and interferon-γ; thus, dietary interventions have been proposed to decrease *IL-18* levels in obese women resulting in weight loss as an important intervention to reduce *IL-18* levels (Esposito et al., 2002). In our study, *IL-18* is also enriched in the “role of *IFN-beta* in the inhibition of T helper 1 (Th1) cell differentiation in multiple sclerosis” pathway, and in this pathway, *IL-18* plays a fundamental role in the differentiation of Th1 cells, which initiate signaling pathways that lead to Th1 cell differentiation and *IFN-gamma* production (Codarri et al., 2010). Multiple sclerosis is an autoimmune disease mediated by autoreactive Th1 that causes damage to the myelin sheath upon entering the central nervous system (Hemmer et al., 2002). *IL-18* was upregulated in the SOY group with possible relation to a pro-inflammatory condition, and on the other hand, the atherogenic index showed no difference between the diets.

With the differential expression between the two comparisons, we also identified common genes such as *CYP7A1* DEG and *PLA2G2D*, which have a function related to the conversion of cholesterol into bile acids and related to the production of lysophospholipids and free FA, respectively. *PLA2G2D* was negatively associated with serum triglyceride and cholesterol concentrations and fractions in the study of Ossabaw pigs. In the study, the pigs were fed a diet high in saturated fat, cholesterol, and refined carbohydrates and low in fiber, compared to pigs fed a diet high in unsaturated fat (oils canola, soy, and corn), unrefined carbohydrates, fruit/vegetables and fiber, and low cholesterol (Ye et al., 2021), demonstrating that the oil sources in pigs’ diets have altered relevant genes. Studies relating the FA profile and differential gene expression are still limited in pigs, and new approaches are needed. Other studies have already emphasized the importance of studying the porcine transcriptome in the study of genes regulating lipid metabolism due to the similarities between pigs and humans (Corominas et al., 2013). The lipid and FA compositions of meat have an important impact on human health in the modulation of genes involved in diseases, such as obesity, diabetes, and neurodegenerative diseases. In this study, we highlight the main genes involved in metabolic and disease pathways regulated by FA in the liver transcriptome. Thus, when we use the human species as a background list for pathway enrichment, we observe mechanisms that can have effects with modifications arising from the diet with different oils. More studies are needed to analyze the FA that specifically modulates each of the genes.

## 5 Conclusion

In this study, we identified relevant changes in the transcriptome profiles and FA deposits in the liver, resulting from feeding different diets containing SOY, CO, or FO, which contain different FA profiles. Between the CO and SOY comparison, despite similar FAs, relevant differences were identified in gene expression. Between SOY and FO, FO presented a greater amount of total n-3 and a consequent reduction in the n-6:n-3 ratio. Pathways were enriched in signaling pathways related to metabolism and metabolic and neurodegenerative diseases. Our results help understand the effects of different oil sources on the behavior of genes with differential expression in metabolic pathways and gene networks in the liver. The identified genes and signaling pathways play an essential role in affecting the liver, and further studies should be performed to elucidate the mechanisms.

## Data Availability

The datasets presented in this study can be found in online repositories. The names of the repository/repositories and accession number(s) can be found in the article/Supplementary Material.
