# Supplementary material for: RNA-seq transcriptome profiling of pigs’ liver in response to diet with different sources of fatty acids
Source: Front Genet. 2023 Jan 25;14:1053021. doi: 10.3389/fgene.2023.1053021 (PMC9936315; doi:10.3389/fgene.2023.1053021)
Supplement: Supplementary file 1 [file DataSheet1.zip › Supplementary Images.pdf]

Supplementary Material

1.1 Supplementary Figures

(A)

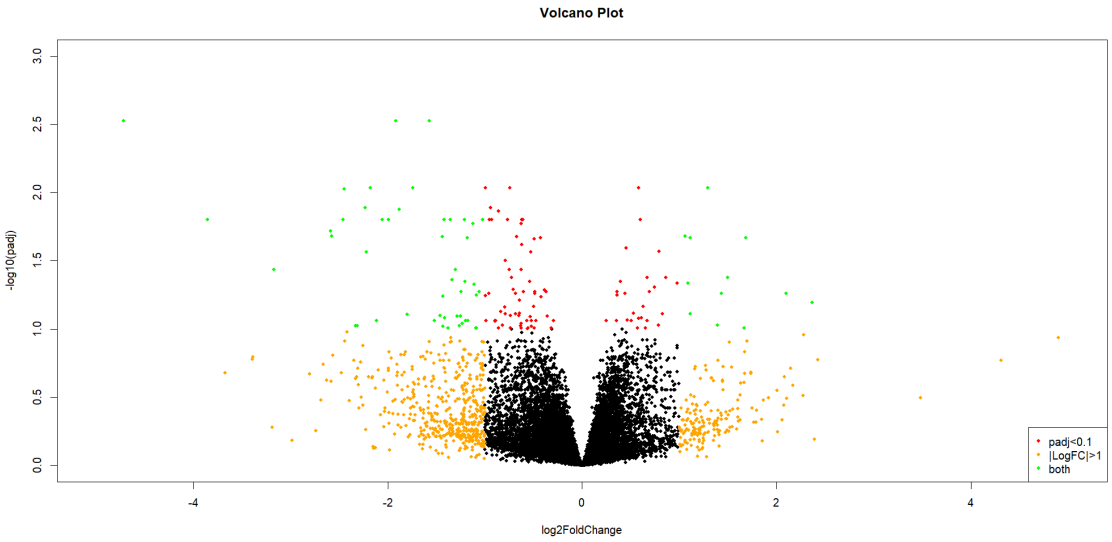

(B)

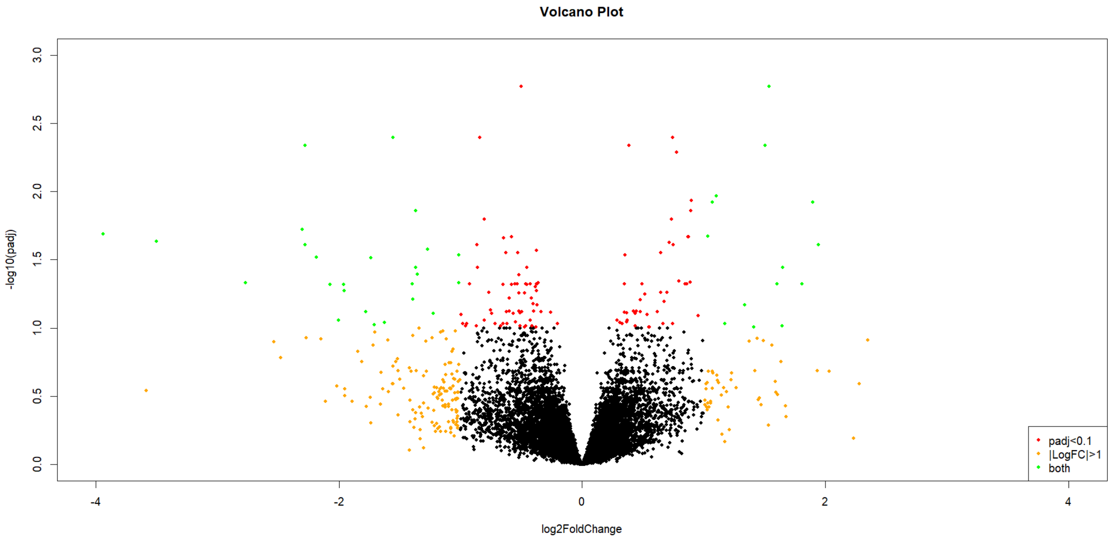

(C)

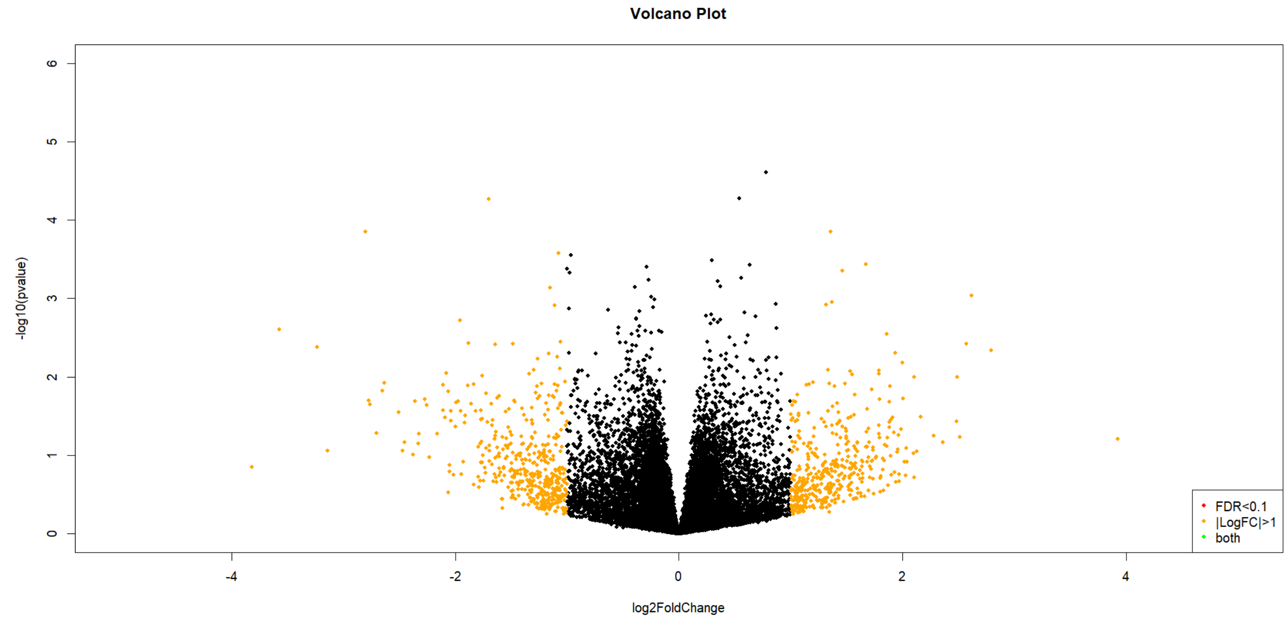

Supplementary Figure 1: Volcano plot of log2-fold change (x-axis) versus -log10FDR-corrected p-value in RNA-Seq data from liver tissue (A) CO *vs* SOY and (B) SOY *vs* FO and (C) CO *vs* FO.

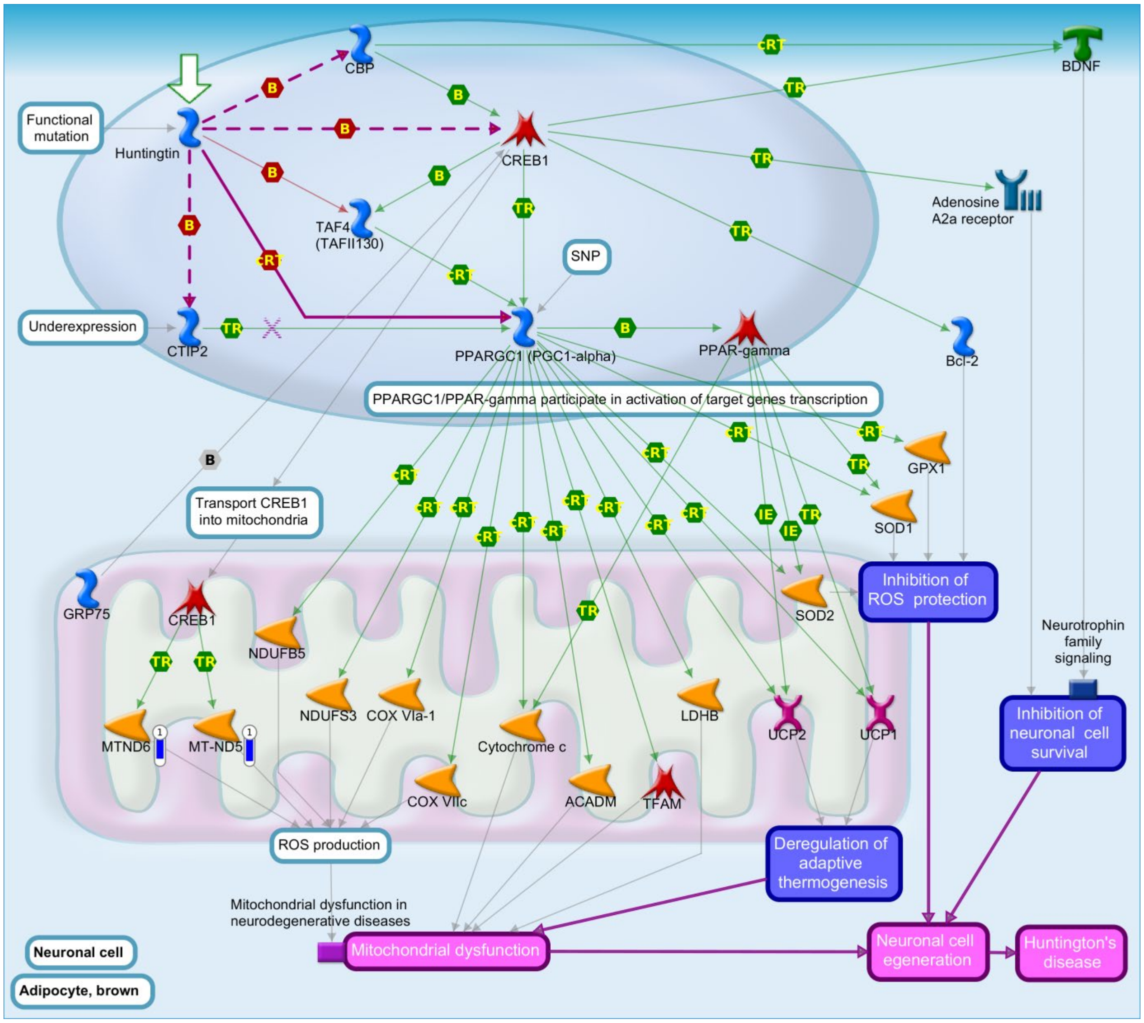

Supplementary Figure 2: *CREB*1-dependent transcription deregulation in Huntington's Diseases pathway map by MetaCore software (*p*-value <0.10) from the list of differentially expressed genes (FDR 10%) in the liver immunocastrated male pigs fed with different oils in the diet (3.0 % canola oil and 3.0 % soybean oil). The blue thermometer indicates that the DEG is down-regulated (log2-fold change -1 and log2-fold change -0.93) in the diet with 3.0 % of canola oil (CO). Purple lines indicate enhances in diseases and purple dotted line emerges in diseases. Green arrows indicate positive interaction and gray arrows indicate unspecified interaction. For a detailed definition, see https://portal.genego.com/legends/MetaCoreQuickReferenceGuide.pdf.

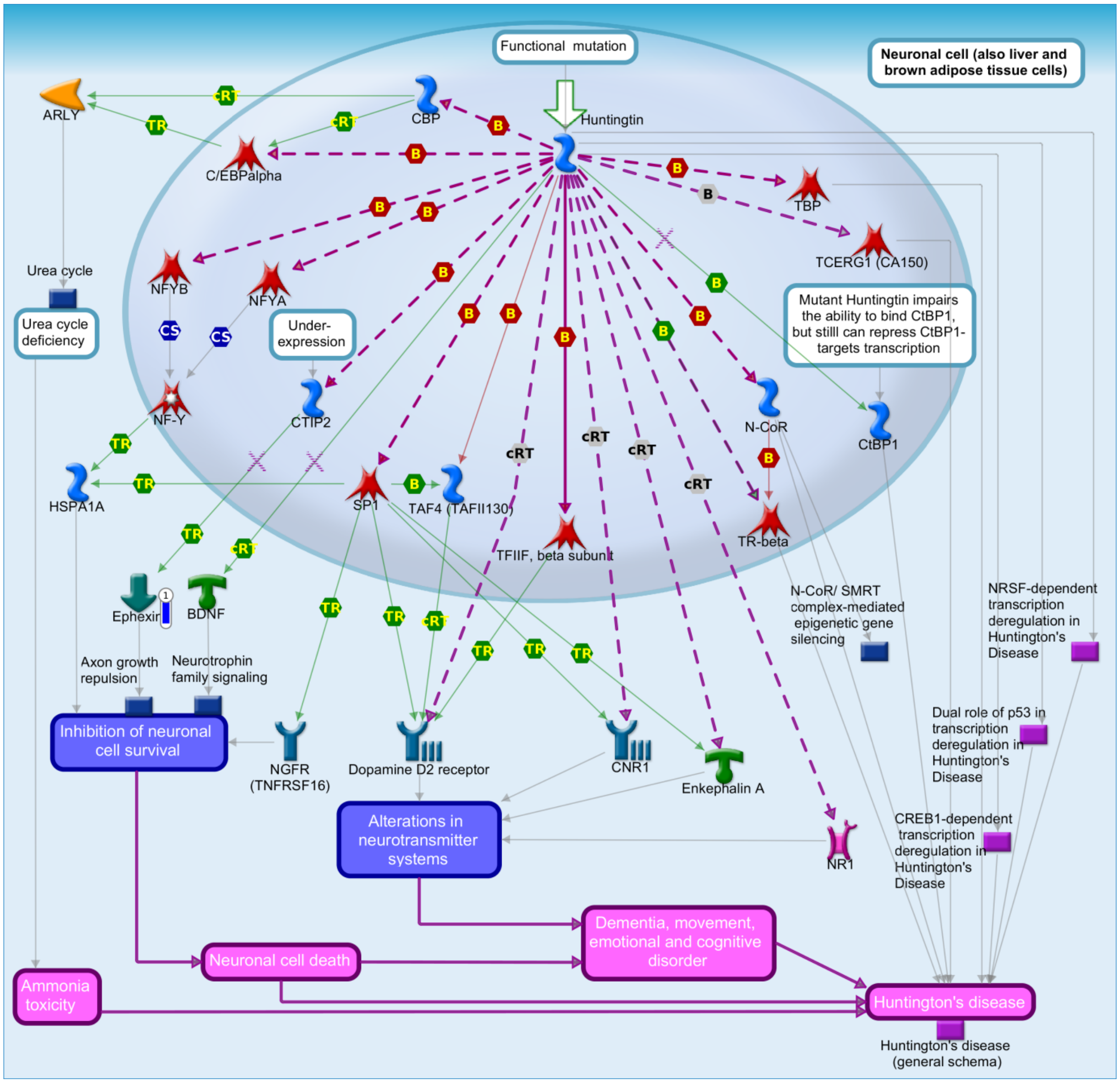

Supplementary Figure 3: Huntingtin-depended transcription deregulation in Huntington's Disease pathway map by MetaCore software (p-value < 0.10) from the list of differentially expressed genes (FDR 10%) in the liver tissue immunocastrated male pigs fed with different oils in the diet (3.0 % canola oil and 3.0 % soybean oil). The blue thermometer indicates that the DEG is down-regulated (log2-fold change -0.6) in the diet with 3.0 % of canola oil (CO). Purple lines indicate enhances in diseases and purple dotted line emerges in diseases. Green arrows indicate positive interaction and gray arrows indicate unspecified interaction. For a detailed definition, see https://portal.genego.com/legends/MetaCoreQuickReferenceGuide.pdf.

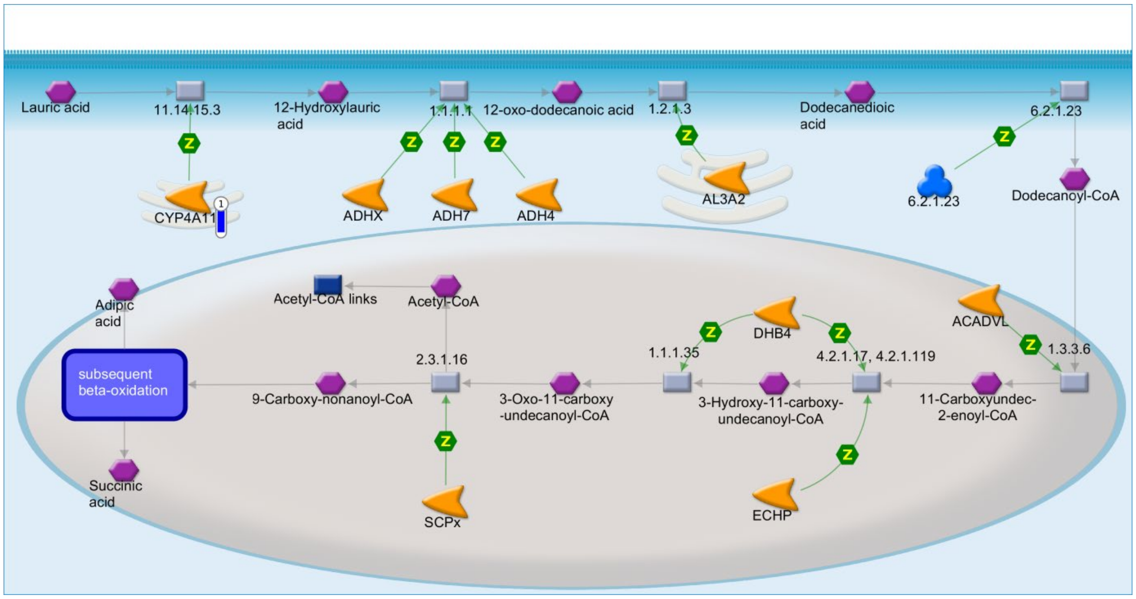

Supplementary Figure 4: Fatty Acid Omega Oxidation pathway map by MetaCore software (*p*-value <0.10) from the list of differentially expressed genes (FDR 10%) in the liver tissue of pigs fed with different oils in the diet (3.0 % canola oil and 3.0 % soybean oil). The blue thermometer indicates that the DEG is down-regulated (log2-fold change -1.25) in the diet with 3.0 % of canola oil (CO). Green arrows indicate positive interaction and gray arrows indicate unspecified interaction. For a detailed definition, see https://portal.genego.com/legends/MetaCoreQuickReferenceGuide.pdf.

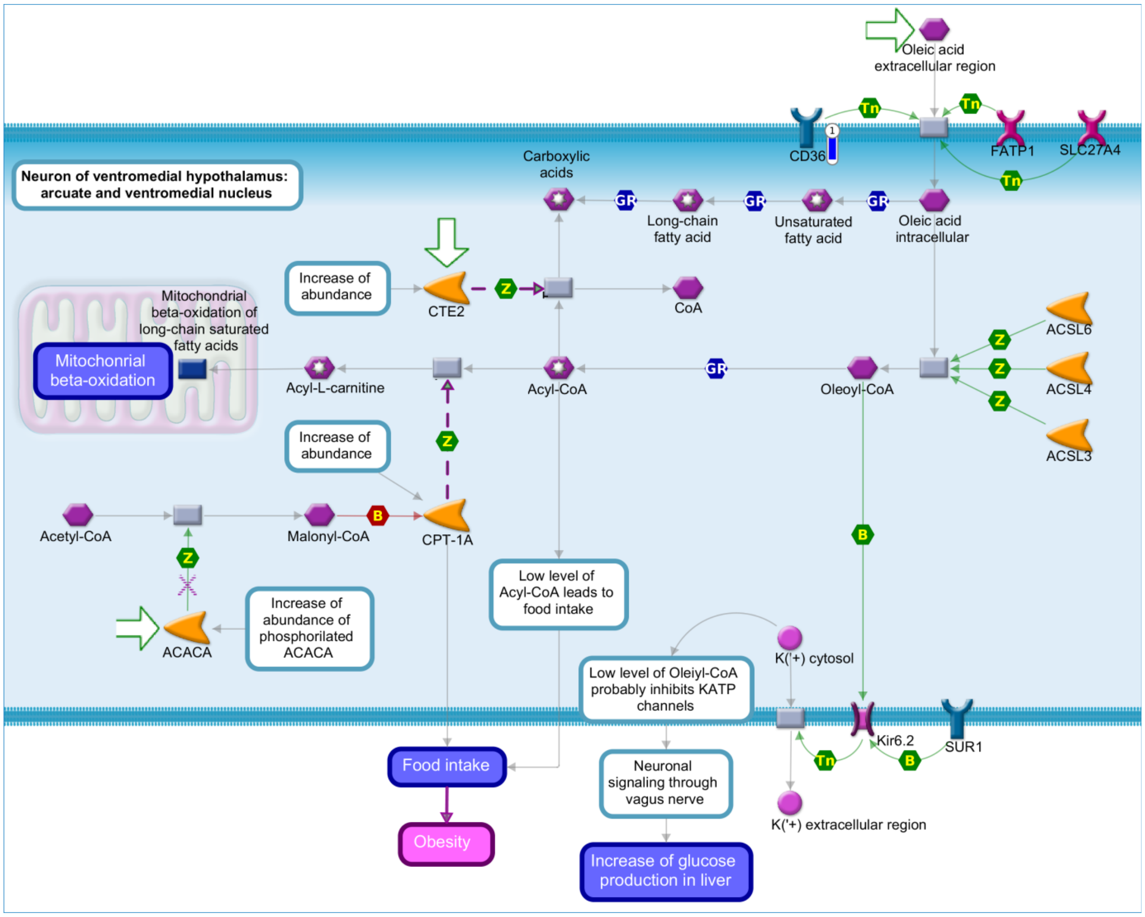

Supplementary Figure 5: Putative pathways of Oleic acid sensing in ventromedial hypothalamus in obesity (rodent model) pathway map by MetaCore software (*p*-value <0.10) from the list of differentially expressed genes (FDR 10%) in the liver tissue of pigs fed with different oils in the diet (3.0 % canola oil and 3.0 % soybean oil). The blue thermometer indicates that the DEG is down-regulated (log2-fold change -0.48) in the diet with 3.0 % of canola oil (CO). Purple lines indicate enhances in diseases and purple dotted line emerges in diseases. Green arrows indicate positive interaction and gray arrows indicate unspecified interaction. For a detailed definition, see https://portal.genego.com/legends/MetaCoreQuickReferenceGuide.pdf.

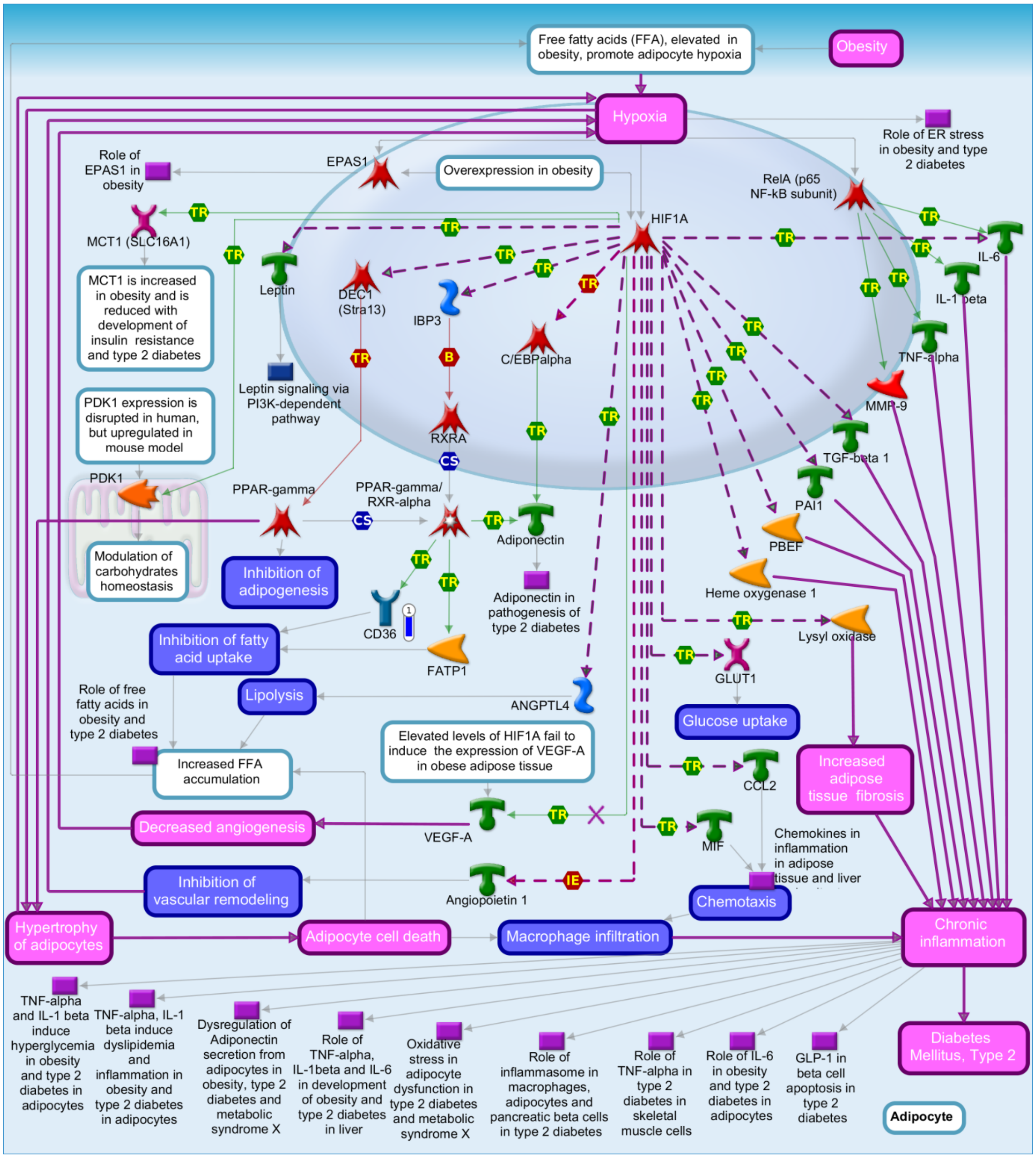

Supplementary Figure 6: Role of adipose tissue hypoxia in obesity and type 2 diabetes pathway map by MetaCore software (*p*-value <0.10) from the list of differentially expressed genes (FDR 10%) in the liver tissue of pigs fed with different oils in the diet (3.0 % canola oil and 3.0 % soybean oil). The blue thermometer indicates that the DEG is down-regulated (log2-fold change -0.48) in the diet with 3.0 % of canola oil (CO). Purple lines indicate enhances in diseases and purple dotted line emerges in diseases. Green arrows indicate positive interaction and gray arrows indicate unspecified interaction. For a detailed definition, see https://portal.genego.com/legends/MetaCoreQuickReferenceGuide.pdf.

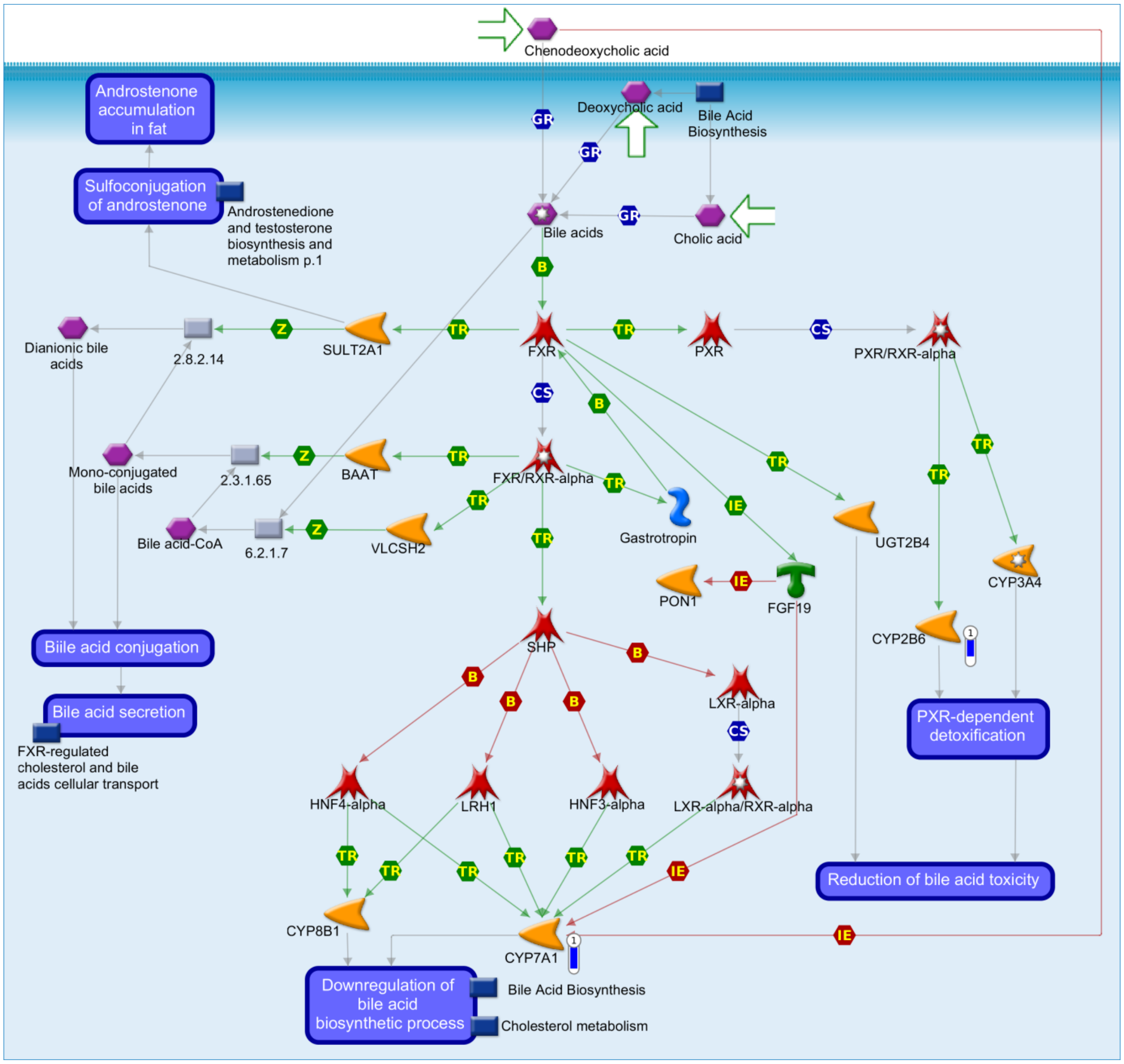

Supplementary Figure 7: Regulation of lipid metabolism FXR-dependent negative-feedback regulation of bile acids concentration pathway map by MetaCore software (p-value <0.10) from the list of differentially expressed genes (FDR 10%) in the liver tissue of pigs fed with different oils in the diet (3.0 % soybean oil and 3.0 % fish oil). The blue thermometer indicates that the DEG is down-regulated in the diet with 3.0 % of soybean oil (SOY). Green arrows indicate positive interaction and gray arrows indicate unspecified interaction. For a detailed definition, see https://portal.genego.com/legends/MetaCoreQuickReferenceGuide.pdf.

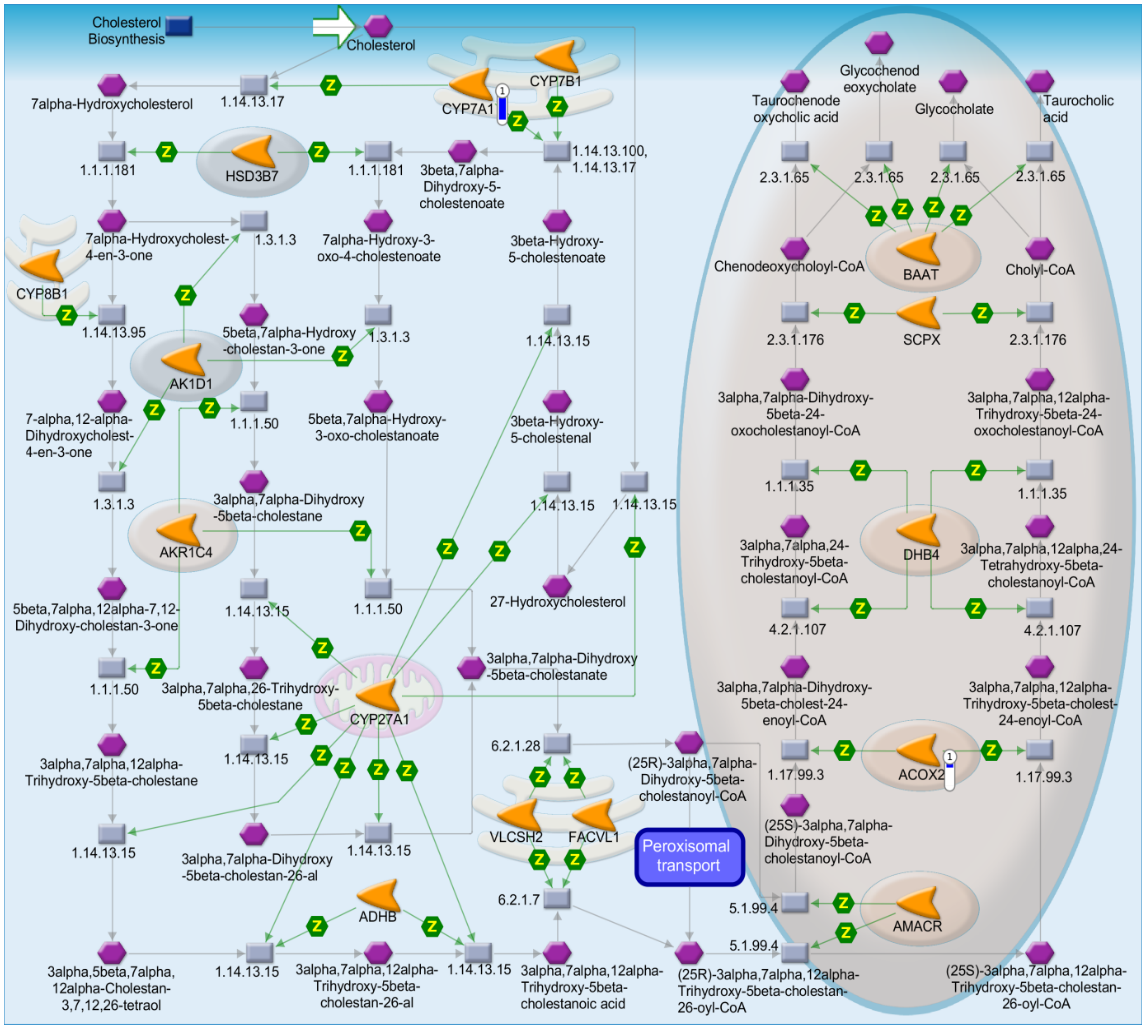

Supplementary Figure 8: Cholesterol metabolism pathway map by MetaCore software (*p*-value <0.10) from the list of differentially expressed genes (FDR 10%) in the liver tissue of pigs fed with different oils in the diet (3.0 % soybean oil and 3.0 % fish oil). The blue thermometer indicates that the DEG is down-regulated in the diet with 3.0 % of soybean oil (SOY). Green arrows indicate positive interaction and gray arrows indicate unspecified interaction. For a detailed definition, see https://portal.genego.com/legends/MetaCoreQuickReferenceGuide.pdf.

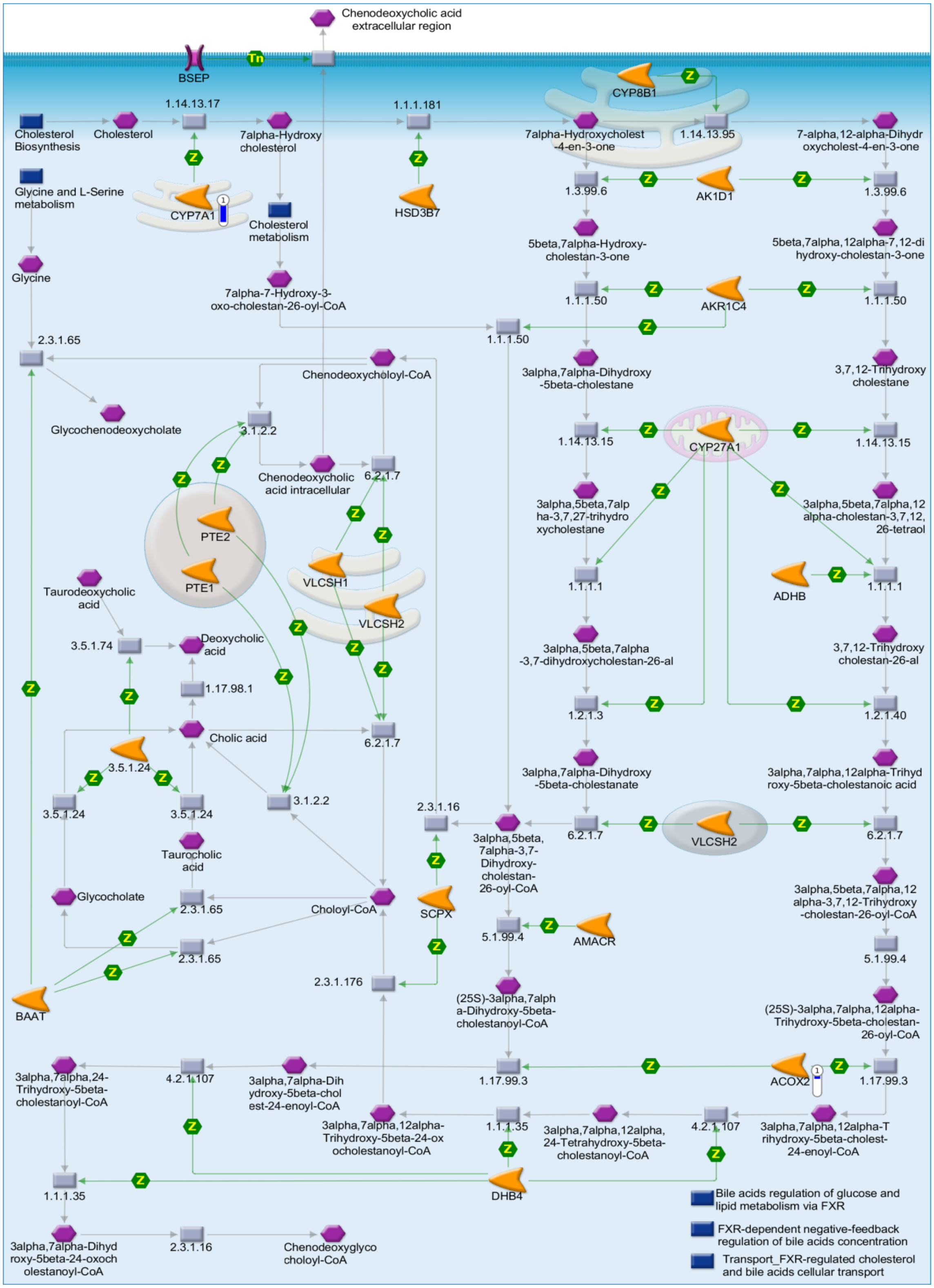

Supplementary Figure 9: Bile Acid Biosynthesis pathway map by MetaCore software (*p*-value <0.10) from the list of differentially expressed genes (FDR 10%) in the liver tissue of pigs fed with different oils in the diet (3.0 % soybean oil and 3.0 % fish oil). The blue thermometer indicates that the DEG is down-regulated in the diet with 3.0 % of soybean oil (SOY). Green arrows indicate

positive interaction and gray arrows indicate unspecified interaction. For a detailed definition, see https://portal.genego.com/legends/MetaCoreQuickReferenceGuide.pdf.

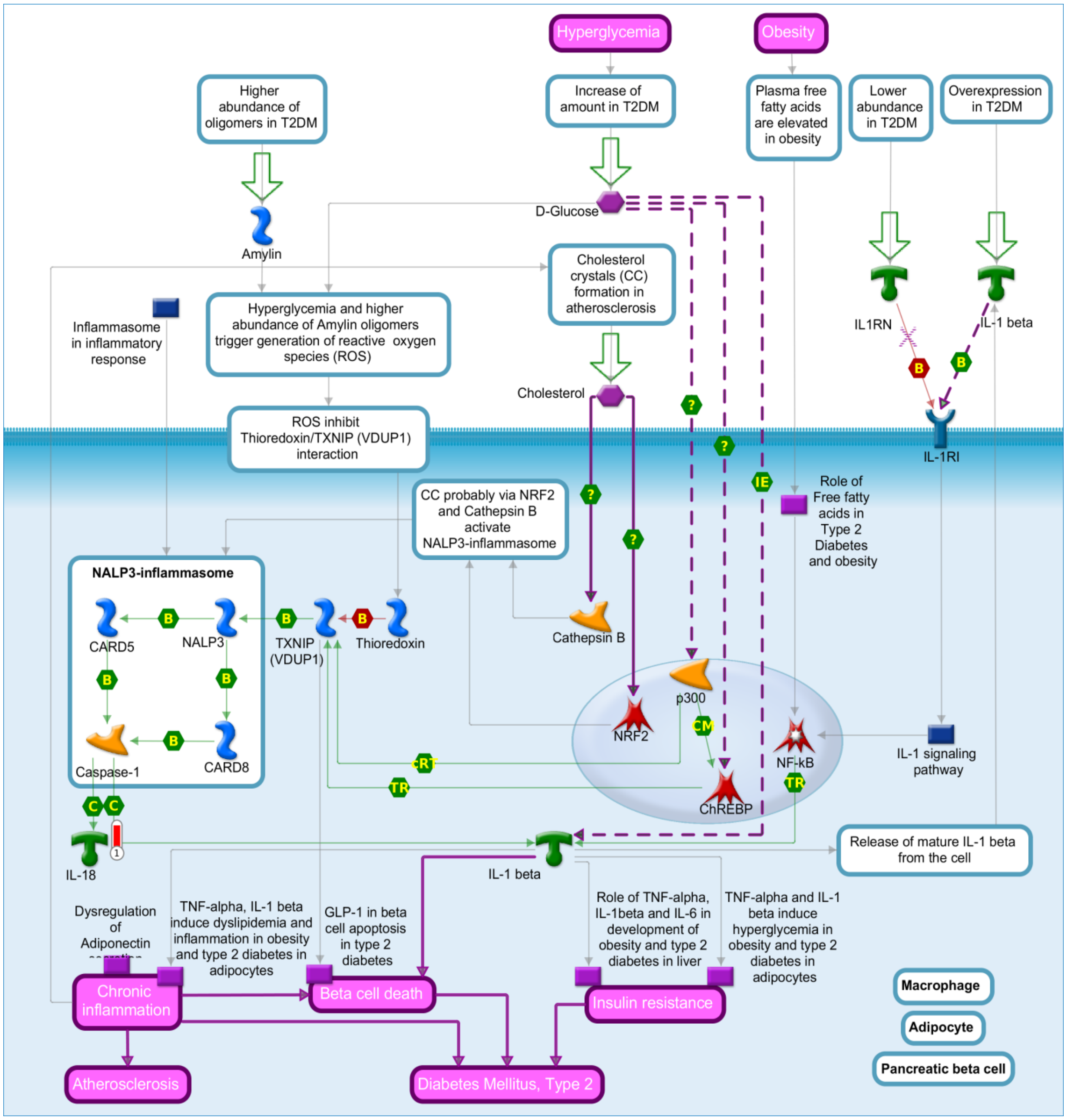

Supplementary Figure 10: Role of inflammasome in macrophages, adipocytes and pancreatic beta cells in type 2 diabetes pathway map by MetaCore software (p-value <0.10) from the list of differentially expressed genes (FDR 10%) in the liver tissue of pigs fed with different oils in the diet (3.0 % soybean oil and 3.0 % fish oil). The blue thermometer indicates that the DEG is down-regulated in the diet with 3.0 % of soybean oil (SOY). Green arrows indicate positive interaction and gray arrows indicate unspecified interaction. For a detailed definition, see https://portal.genego.com/legends/MetaCoreQuickReferenceGuide.pdf.

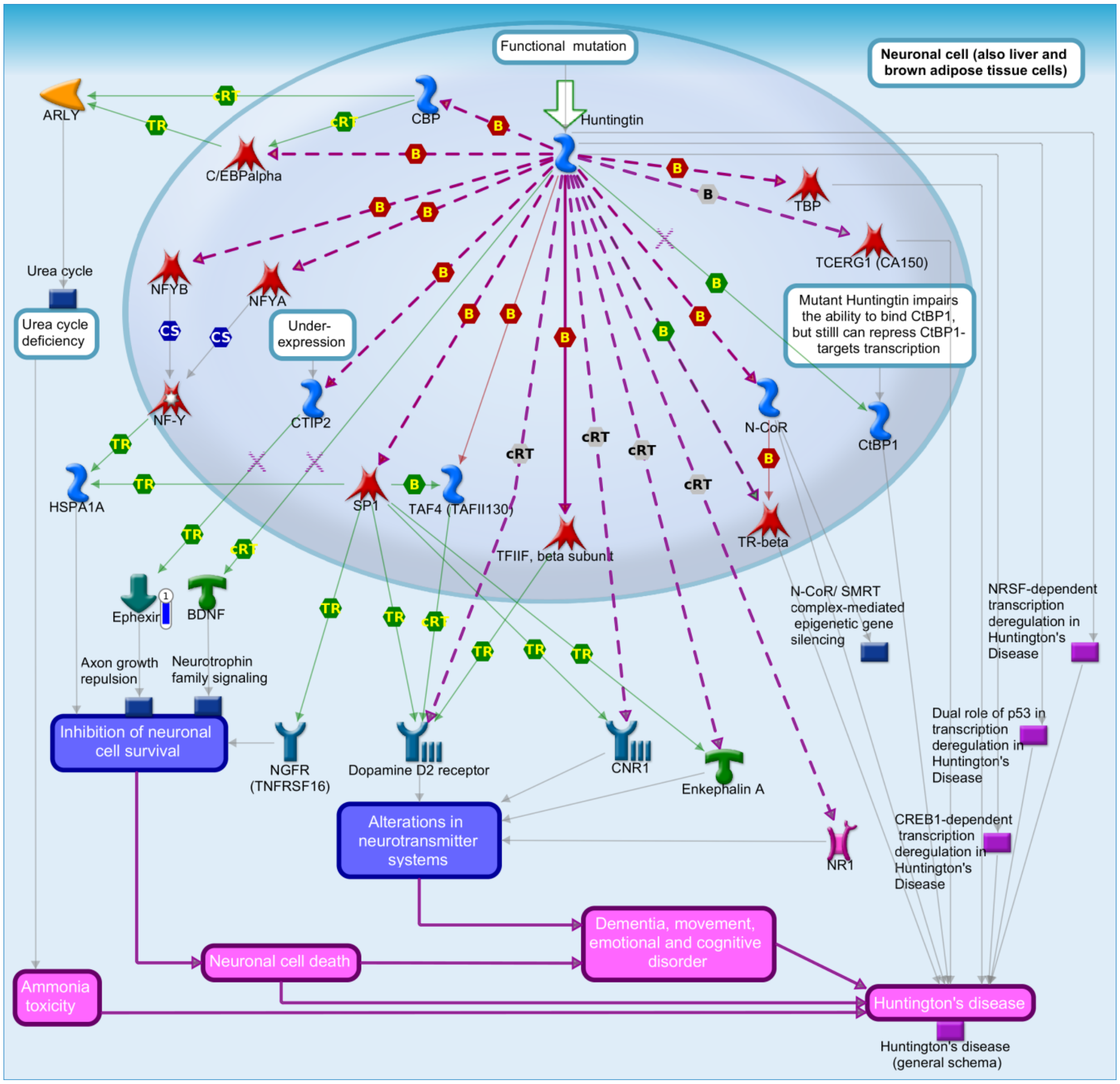

Supplementary Figure 11: Huntingtin-depended transcription deregulation in Huntington's Disease pathway map by MetaCore software (*p*-value <0.10) from the list of differentially expressed genes (FDR 10%) in the liver tissue of pigs fed with different oils in the diet (3.0 % soybean oil and 3.0 % fish oil). The blue thermometer indicates that the DEG is down-regulated in the diet with 3.0 % of soybean oil (SOY). Green arrows indicate positive interaction and gray arrows indicate unspecified interaction. For a detailed definition, see https://portal.genego.com/legends/MetaCoreQuickReferenceGuide.pdf.

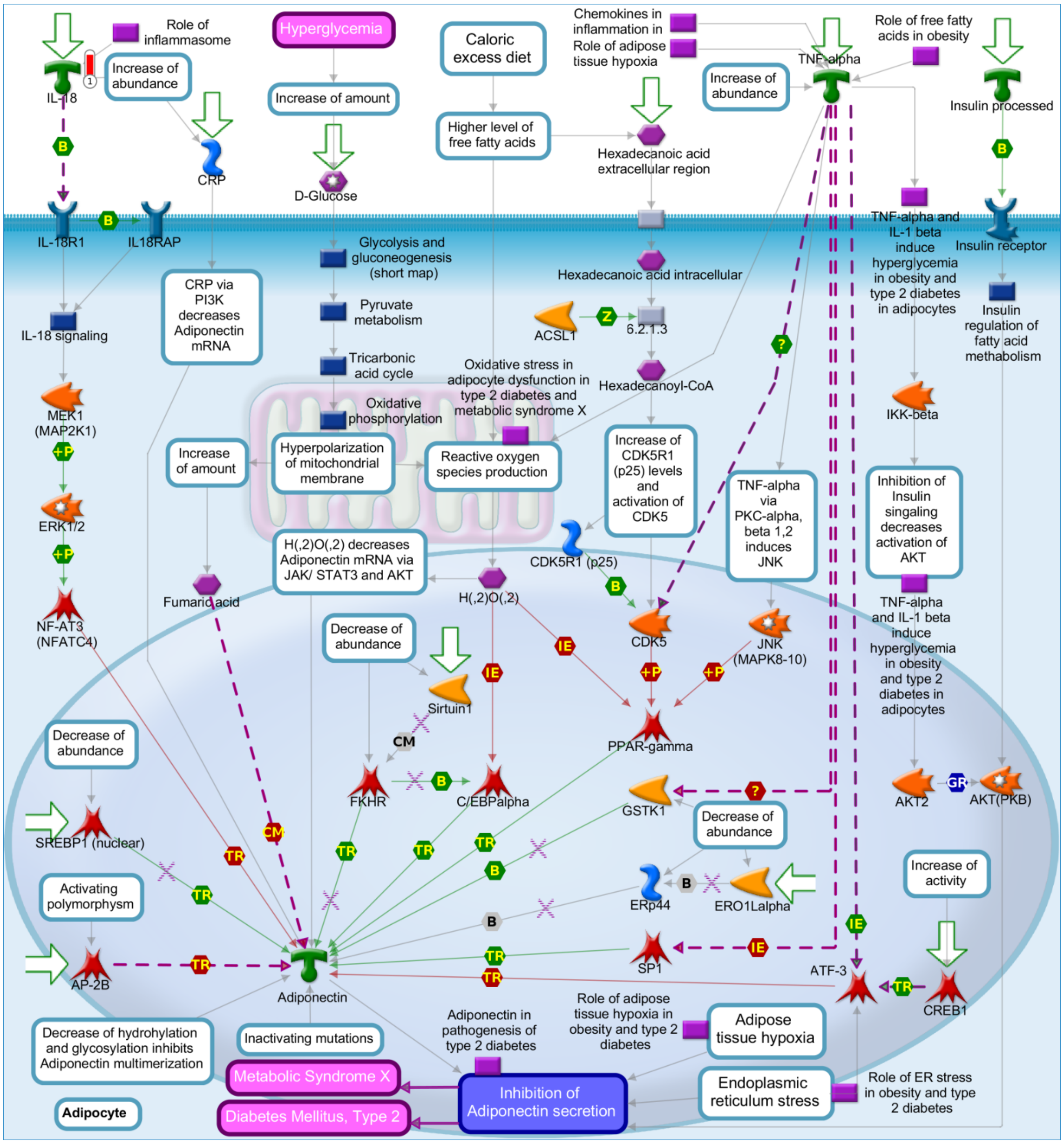

Supplementary Figure 12: Role of IFN-beta in inhibition of Th1 cell differentiation in multiple sclerosis pathway map by MetaCore software (p-value <0.10) from the list of differentially expressed genes (FDR 10%) in the liver tissue of pigs fed with different oils in the diet (3.0 % soybean oil and 3.0 % fish oil). The blue thermometer indicates that the DEG is down-regulated in the diet with 3.0 % of soybean oil (SOY). Green arrows indicate positive interaction and gray arrows indicate unspecified interaction. For a detailed definition, see https://portal.genego.com/legends/MetaCoreQuickReferenceGuide.pdf.
