# Supplementary material for: RNA-seq transcriptome profiling of pigs’ liver in response to diet with different sources of fatty acids
Source: Front Genet. 2023 Jan 25;14:1053021. doi: 10.3389/fgene.2023.1053021 (PMC9936315; doi:10.3389/fgene.2023.1053021)
Supplement: Supplementary file 1 [file DataSheet1.zip › Supplementary Tables 1- 4.docx]

**Supplementary Table 1** Composition of the experimental diets^1^ (as-fed basis)

|  | Grower I | |  | Grower II | |  | Finisher I | |  | Finisher II | |  | Finisher III | |  | Finisher IV | |
| --- | --- | --- | --- | --- | --- | --- | --- | --- | --- | --- | --- | --- | --- | --- | --- | --- | --- |
|  | (day 0 to 21) | |  | (day 21 to 42) | |  | (day 42 to 56) | |  | (day 56 to 63) | |  | (day 63 to 70) | |  | (day 70 to 98) | |
| Item |  |  |  |  |  |  |  |  |  |  |  |  |  |  |  |  |  |
| Ingredient, % |  |  |  |  |  |  |  |  |  |  |  |  |  |  |  |  |  |
| Corn, 7.5% CP^2^ |  | 61.88 |  |  | 64.71 |  |  | 67.54 |  |  | 68.04 |  |  | 68.00 |  |  | 68.50 |
| Soybean meal, 46% CP |  | 28.42 |  |  | 26.29 |  |  | 23.46 |  |  | 23.46 |  |  | 23.02 |  |  | 23.02 |
| Meat and bone meal, 44% CP |  | 3.00 |  |  | 3.00 |  |  | 3.00 |  |  | 3.00 |  |  | 3.00 |  |  | 3.00 |
| Fat source |  | 3.00 |  |  | 3.00 |  |  | 3.00 |  |  | 3.00 |  |  | 3.00 |  |  | 3.00 |
| Dicalcium phosphate |  | 0.56 |  |  | 0.57 |  |  | 0.27 |  |  | 0.27 |  |  | 0.27 |  |  | 0.27 |
| Limestone |  | 0.42 |  |  | 0.38 |  |  | 0.84 |  |  | 0.75 |  |  | 0.84 |  |  | 0.69 |
| Salt |  | 0.50 |  |  | 0.50 |  |  | 0.50 |  |  | 0.50 |  |  | 0.50 |  |  | 0.50 |
| Vitamin-mineral premix^3^ |  | 1.61 |  |  | 1.08 |  |  | 1.01 |  |  | 0.60 |  |  | 1.01 |  |  | 0.65 |
| L-Lysine.HCl |  | 0.35 |  |  | 0.29 |  |  | 0.25 |  |  | 0.25 |  |  | 0.20 |  |  | 0.20 |
| DL-Methionine |  | 0.11 |  |  | 0.08 |  |  | 0.04 |  |  | 0.04 |  |  | 0.02 |  |  | 0.03 |
| L-Threonine |  | 0.15 |  |  | 0.11 |  |  | 0.09 |  |  | 0.09 |  |  | 0.06 |  |  | 0.07 |
| L-Tryptophan |  | 0.01 |  |  | - |  |  | - |  |  | - |  |  | - |  |  | - |
| Ractopamine.HCl, 2% |  | - |  |  | - |  |  | - |  |  | - |  |  | 0.08 |  |  | 0.08 |
| Calculated composition^4^ |  |  |  |  |  |  |  |  |  |  |  |  |  |  |  |  |  |
| Metabolizable energy, Mcal/kg |  | 3.36 |  |  | 3.36 |  |  | 3.36 |  |  | 3.36 |  |  | 3.35 |  |  | 3.36 |
| SID^5^ Lysine, % |  | 1.15 |  |  | 1.05 |  |  | 0.95 |  |  | 0.95 |  |  | 0.90 |  |  | 0.90 |
| SID Methionine + Cysteine, % |  | 0.62 |  |  | 0.57 |  |  | 0.51 |  |  | 0.51 |  |  | 0.49 |  |  | 0.49 |
| SID Threonine, % |  | 0.75 |  |  | 0.68 |  |  | 0.63 |  |  | 0.63 |  |  | 0.59 |  |  | 0.59 |
| SID Tryptophan, % |  | 0.22 |  |  | 0.20 |  |  | 0.18 |  |  | 0.18 |  |  | 0.18 |  |  | 0.18 |
| Calcium, % |  | 0.84 |  |  | 0.81 |  |  | 0.80 |  |  | 0.77 |  |  | 0.80 |  |  | 0.75 |
| Available Phosphorous, % |  | 0.42 |  |  | 0.42 |  |  | 0.34 |  |  | 0.34 |  |  | 0.34 |  |  | 0.34 |
| Analyzed composition, % |  |  |  |  |  |  |  |  |  |  |  |  |  |  |  |  |  |
| CP |  | 19.64 |  |  | 18.71 |  |  | 17.58 |  |  | 17.59 |  |  | 17.33 |  |  | 17.35 |
| Ether extract |  | 5.49 |  |  | 5.53 |  |  | 6.57 |  |  | 5.59 |  |  | 5.61 |  |  | 5.61 |

^1^The added oil diets consisted of 3% soybean oil (SOY), or canola oil (CO), or fish oil (FO).

^2^CP = crude protein.

^3^Provided per kilogram of diet: 6.500 UI vitamin A; 1.800 UI vitamin D_3_; 30 UI vitamin E; 2 mg vitamin K_3_; 1.2 mg vitamin B_1_; 3.4 mg vitamin B_2_; 2.0 mg vitamin B_6_; 125 mg Cu; 80 mg Fe; 40 mg Mn; 0.35 mg Se; 1.25 mg Zn.

^4^Calculated according to Rostagno et al. (2011).

^5^SID = standardized ileal digestible.

**Supplementary Table 2** Analyzed fatty acid profile of grower diets^1^ (as-fed basis)

|  | Grower I (day 0 to 21) | | | | |  | Grower II (day 21 to 42) | | | | |
| --- | --- | --- | --- | --- | --- | --- | --- | --- | --- | --- | --- |
| Fatty acid, % | SO | CO | FO |  |  | | | SO | CO | FO |  |
| Saturated fatty acid (SFA) |  |  |  |  |  | | |  |  |  |  |
| Myristic acid (C14:0) | 1.85 | ND | 0.24 |  |  | | | 0.31 | 0.16 | 1.53 |  |
| Palmitic acid (C16:0) | 12.76 | 10.82 | 20.36 |  |  | | | 14.37 | 10.54 | 18.34 |  |
| Margaric acid (C17:0) | ND | ND | ND |  |  | | | ND | ND | ND |  |
| Stearic acid (C18:0) | 2.44 | 3.83 | 5.01 |  |  | | | 4.50 | 3.13 | 4.52 |  |
| Arachidic acid (C20:0) | ND | 0.57 | 0.35 |  |  | | | 0.45 | 0.61 | 0.44 |  |
| Behenic acid (C22:0) | ND | 0.26 | 0.16 |  |  | | | 0.24 | 0.23 | ND |  |
| Monounsaturated fatty acid (MUFA) |  |  |  |  |  | | |  |  |  |  |
| Palmitoleic acid (C16:1) | ND | ND | 3.45 |  |  | | | 0.26 | 0.19 | 2.90 |  |
| Oleic acid (C18:1 n-9) | 23.27 | 47.52 | 33.16 |  |  | | | 31.92 | 49.72 | 37.65 |  |
| Eicosenoic acid (C20:1 n-9) | ND | 0.64 | 0.88 |  |  | | | 0.21 | 0.69 | 0.85 |  |
| Polyunsaturated fatty acid (PUFA) |  |  |  |  |  | | |  |  |  |  |
| Linoleic acid (C18:2 n-6) | 55.65 | 32.02 | 29.94 |  |  | | | 44.80 | 33.00 | 30.15 |  |
| Alpha-linolenic acid (C18:3 n-3) | 5.85 | 4.31 | 1.62 |  |  | | | 2.95 | 1.74 | 1.47 |  |
| Eicosapentaenoic acid (C20:5 n-3) | ND | ND | 1.06 |  |  | | | ND | ND | 0.83 |  |
| Docosahexaenoic acid (C22:6 n-3) | ND | ND | 1.78 |  |  | | | ND | ND | 1.33 |  |
| Total SFA | 17.05 | 15.48 | 26.12 |  |  | | | 19.87 | 14.67 | 24.83 |  |
| Total MUFA | 23.27 | 48.16 | 37.49 |  |  | | | 32.39 | 50.6 | 41.4 |  |
| Total PUFA | 61.5 | 36.33 | 34.4 |  |  | | | 47.75 | 34.74 | 33.78 |  |
| PUFA:SFA ratio^3^ | 3.61 | 2.35 | 1.32 |  |  | | | 2.40 | 2.37 | 1.36 |  |

^1^The added oil diets consisted of 3.0% soybean oil (SOY), or canola oil (CO), or fish oil (FO).

^2^ND = not detectable.

^3^PUFA:SFA ratio = total PUFA/total SFA.

Adapted from Almeida et al. (2021).

**Supplementary Table 3** Analyzed fatty acid profile of finisher diets^1^ (as-fed basis)

|  | Finisher I (day 42 to 56) | | | | |  | Finisher II (day 56 to 63) | | | | |
| --- | --- | --- | --- | --- | --- | --- | --- | --- | --- | --- | --- |
| Fatty acid, % | SO | CO | FO |  |  | | | SO | CO | FO |  |
| Saturated fatty acid (SFA) |  |  |  |  |  | | |  |  |  |  |
| Myristic acid (C14:0) | 0.28 | ND^2^ | ND |  |  | | | ND | 0.16 | 1.75 |  |
| Palmitic acid (C16:0) | 13.82 | 10.64 | 13.72 |  |  | | | 12.59 | 10.06 | 19.30 |  |
| Margaric acid (C17:0) | 0.16 | ND | ND |  |  | | | ND | 0.07 | 0.24 |  |
| Stearic acid (C18:0) | 4.28 | 3.35 | 4.42 |  |  | | | 2.83 | 2.40 | 4.12 |  |
| Arachidic acid (C20:0) | 0.43 | 0.57 | 0.35 |  |  | | | 0.41 | 0.58 | 0.36 |  |
| Behenic acid (C22:0) | 0.19 | ND | 0.13 |  |  | | | 0.21 | 0.25 | ND |  |
| Monounsaturated fatty acid (MUFA) |  |  |  |  |  | | |  |  |  |  |
| Palmitoleic acid (C16:1) | 0.32 | 0.27 | 0.19 |  |  | | | 0.11 | 0.28 | 3.50 |  |
| Oleic acid (C18:1 n-9) | 34.95 | 52.98 | 35.96 |  |  | | | 34.84 | 52.58 | 36.62 |  |
| Eicosenoic acid (C20:1 n-9) | 0.26 | 0.71 | 0.78 |  |  | | | 0.23 | 0.73 | 0.92 |  |
| Polyunsaturated fatty acid (PUFA) |  |  |  |  |  | | |  |  |  |  |
| Linoleic acid (C18:2 n-6) | 42.85 | 30.34 | 43.04 |  |  | | | 46.03 | 31.64 | 28.79 |  |
| Alpha-linolenic acid (C18:3 n-3) | 2.45 | 1.13 | 2.67 |  |  | | | 2.76 | 1.09 | 1.57 |  |
| Eicosapentaenoic acid (C20:5 n-3) | ND | ND | 1.02 |  |  | | | ND | ND | 1.04 |  |
| Docosahexaenoic acid (C22:6 n-3) | ND | ND | 1.73 |  |  | | | ND | ND | 1.79 |  |
| Total SFA | 19.16 | 14.56 | 18.62 |  |  | | | 16.04 | 13.52 | 25.77 |  |
| Total MUFA | 35.53 | 53.96 | 36.93 |  |  | | | 35.18 | 53.59 | 41.04 |  |
| Total PUFA | 45.30 | 31.47 | 48.46 |  |  | | | 48.79 | 32.73 | 33.19 |  |
| PUFA:SFA ratio^3^ | 2.36 | 2.16 | 2.60 |  |  | | | 3.04 | 2.42 | 1.29 |  |

^1^The added oil diets consisted of 3% soybean oil (SO), or canola oil (CO), or fish oil (FO).

^2^ND = not detectable.

^3^PUFA:SFA ratio = total PUFA/total SFA.

Adapted from Almeida et al. (2021).

**Supplementary Table 4** Analyzed fatty acid profile of finisher diets^1^ (as-fed basis)

|  | Finisher III (day 63 to 70) | | | | |  | | Finisher IV (day 70 to 98) | | | |
| --- | --- | --- | --- | --- | --- | --- | --- | --- | --- | --- | --- |
| Fatty acid, % | SO | CO | FO |  |  | | SO | | CO | FO |  |
| Saturated fatty acid (SFA) |  |  |  |  |  | |  | |  |  |  |
| Myristic acid (C14:0) | 0.28 | 0.25 | 1.88 |  |  | | ND | | ND | 1.90 |  |
| Palmitic acid (C16:0) | 13.82 | 10.71 | 20.02 |  |  | | 14.45 | | 10.60 | 20.11 |  |
| Margaric acid (C17:0) | 0.16 | 0.13 | ND |  |  | | ND | | ND | ND |  |
| Stearic acid (C18:0) | 4.28 | 3.61 | 5.06 |  |  | | 4.53 | | 3.39 | 4.73 |  |
| Arachidic acid (C20:0) | 0.43 | 0.54 | ND |  |  | | 0.43 | | 0.58 | ND |  |
| Behenic acid (C22:0) | 0.19 | 0.26 | ND |  |  | | ND | | ND | ND |  |
| Monounsaturated fatty acid (MUFA) |  |  |  |  |  | |  | |  |  |  |
| Palmitoleic acid (C16:1) | 0.32 | 0.24 | 3.52 |  |  | | 0.22 | | 0.39 | 3.61 |  |
| Oleic acid (C18:1 n-9) | 34.95 | 48.67 | 36.77 |  |  | | 35.58 | | 52.26 | 35.44 |  |
| Eicosenoic acid (C20:1 n-9) | 0.26 | 0.62 | ND |  |  | | ND | | 0.71 | 0.90 |  |
| Polyunsaturated fatty acid (PUFA) |  |  |  |  |  | |  | |  |  |  |
| Linoleic acid (C18:2 n-6) | 42.85 | 33.47 | 28.41 |  |  | | 42.56 | | 30.99 | 28.92 |  |
| Alpha-linolenic acid (C18:3 n-3) | 2.45 | 1.51 | 1.52 |  |  | | 2.24 | | 1.07 | 1.55 |  |
| Eicosapentaenoic acid (C20:5 n-3) | ND | ND | 1.05 |  |  | | ND | | ND | 1.07 |  |
| Docosahexaenoic acid (C22:6 n-3) | ND | ND | 1.78 |  |  | | ND | | ND | 1.77 |  |
| Total SFA | 19.16 | 15.5 | 26.96 |  |  | | 19.41 | | 14.57 | 26.74 |  |
| Total MUFA | 35.53 | 49.53 | 40.29 |  |  | | 35.8 | | 53.36 | 39.95 |  |
| Total PUFA | 45.3 | 34.98 | 32.76 |  |  | | 44.8 | | 32.06 | 33.31 |  |
| PUFA:SFA ratio^3^ | 2.36 | 2.26 | 1.22 |  |  | | 2.31 | | 2.20 | 1.25 |  |

^1^The added oil diets consisted of 3.0% soybean oil (SOY) or canola oil (CO), or fish oil (FO).

^2^ND = not detectable.

^3^PUFA:SFA ratio = total PUFA/total SFA.

Adapted from Almeida et al. (2021).
